# Metal ion coordination delays amyloid-β peptide self-assembly by forming an aggregation–inert complex

**DOI:** 10.1074/jbc.RA120.012738

**Published:** 2020-04-02

**Authors:** Cecilia Wallin, Jüri Jarvet, Henrik Biverstål, Sebastian Wärmländer, Jens Danielsson, Astrid Gräslund, Axel Abelein

**Affiliations:** ‡ Department of Biochemistry and Biophysics, The Arrhenius Laboratories, Stockholm University, 106 91 Stockholm, Sweden; § Department of Neurobiology, Care Sciences and Society, Center for Alzheimer Research, Division of Neurogeriatrics, Karolinska Institutet, 141 52 Huddinge, Sweden; ¶ Department of Physical Organic Chemistry, Latvian Institute of Organic Synthesis, Riga LV-1006, Latvia

**Keywords:** protein aggregation, amyloid-beta (AB), amyloid, Alzheimer disease, metal ion-protein interaction, metal, nuclear magnetic resonance (NMR), neurodegeneration, zinc, monovalent ion, silver

## Abstract

A detailed understanding of the molecular pathways for amyloid-β (Aβ) peptide aggregation from monomers into amyloid fibrils, a hallmark of Alzheimer's disease, is crucial for the development of diagnostic and therapeutic strategies. We investigate the molecular details of peptide fibrillization *in vitro* by perturbing this process through addition of differently charged metal ions. Here, we used a monovalent probe, the silver ion, that, similarly to divalent metal ions, binds to monomeric Aβ peptide and efficiently modulates Aβ fibrillization. On the basis of our findings, combined with our previous results on divalent zinc ions, we propose a model that links the microscopic metal-ion binding to Aβ monomers to its macroscopic impact on the peptide self-assembly observed in bulk experiments. We found that substoichiometric concentrations of the investigated metal ions bind specifically to the N-terminal region of Aβ, forming a dynamic, partially compact complex. The metal-ion bound state appears to be incapable of aggregation, effectively reducing the available monomeric Aβ pool for incorporation into fibrils. This is especially reflected in a decreased fibril-end elongation rate. However, because the bound state is significantly less stable than the amyloid state, Aβ peptides are only transiently redirected from fibril formation, and eventually almost all Aβ monomers are integrated into fibrils. Taken together, these findings unravel the mechanistic consequences of delaying Aβ aggregation *via* weak metal-ion binding, quantitatively linking the contributions of specific interactions of metal ions with monomeric Aβ to their effects on bulk aggregation.

## Introduction

Association and dissociation of protein–protein and metal–protein complexes are highly relevant in biological systems, both for function and misfunction related to diseases (1). The interplay between proteins and metal ions, including the stability/lifetime of such complexes, might be key for an understanding of the disease-associated molecular processes. One central event in Alzheimer's disease is the formation of cerebral senile plaques consisting of aggregated amyloid-β (Aβ)^2^ peptides (2, 3). The self-assembly of the Aβ peptide can be described as a nucleation-dependent process, and detailed information about the fibrillization and nucleation mechanisms may be of importance for an understanding of the disease pathology (4–7). Metal ions have been shown to be potent modulators of Aβ aggregation (8–10), exhibiting specific binding to the intrinsically disordered Aβ monomer (8–14). Their small size and variable charge makes them suitable agents to elucidate the effect of modulating the Aβ monomer structure upon metal-ion binding on the overall Aβ fibrillization process. In general, metal ions such as copper, zinc, and iron are implicated in Alzheimer's disease pathology (15–17) and are also abundantly sequestered in senile plaques (18), but also other metal ions may take key roles, *e.g.* through exogenous contamination and competition with the same binding ligands as endogenous metal ions (13, 19–23). Here we use metal ions in substoichiometric concentrations as amyloid aggregation modulators to disturb the aggregating system to obtain further insights into the fundamental self-assembly mechanisms.

The monomeric Aβ peptides are predominantly unstructured (24, 25), yet are prone to self-assembly into larger aggregates, and eventually they form fibrils with a stable cross-β structure (26, 27). The main coordinating metal ligands in the Aβ peptide are in the hydrophilic N-terminal part (12, 28–30). Findings from NMR and molecular modeling studies suggest one main binding mode consisting of three histidines and the N-terminal aspartic acid (28–30), where Glu^11^ is an alternative potential fourth ligand (20). A specific metal-binding mode affects the structure of the Aβ peptide and hence its properties. The net charge of the Aβ peptide at physiological pH is approximately −3, and interactions with cations decrease the net charge and the intra- and interpeptide electrostatic repulsions. The metal ion effects on the Aβ aggregation kinetics are concentration-dependent. Superstoichiometric concentrations of Cu(II) and Zn(II) easily induce rapid formation of amorphous aggregates without amyloid structures, where the metal ion might bind to a second binding site and possibly bridge between Aβ peptides (9, 31, 32). In contrast, substoichiometric Cu(II) (33, 34) or Zn(II) concentrations retard the overall fibrillization process, and our previous study on Zn(II) revealed the specific reduction of the fibril-end elongation (14). In addition to Cu(II) and Zn(II), several other transition metal ions bind to the Aβ peptide, competing for similar ligands with slightly different coordination modes (13, 19–21).

Silver ions are well-known protein-binding ions and substantially used in the past as a protein-staining agent (35, 36). In addition to changes in charge, the ionic radii of Ag(I) and divalent metal ions differ, together with small deviations in preferred binding ligands and coordination geometry (Table S1). Both *in vitro* and *in vivo* studies showed Cu(I) replacement by Ag(I) ions in copper-containing proteins (37–39). Ag(I) ions exhibit a larger Pauling radius and a smaller charge density than Cu(I) ions (Table S1), but because of the same electric charge number, the more stable Ag(I) ions have been used as a probe for the readily redox-active Cu(I) ions in *in vitro* studies (40–42). In fact, a recent study investigated Cu(I) and Ag(I) binding toward the model peptide Aβ_16_ and showed similar but slightly different binding modes (42). Furthermore, silver ions are not paramagnetic, in contrast to Cu(II), and are thus a useful substitute for interaction studies using NMR.

Studying misfolding and aggregation of amyloidogenic proteins by varying the experimental conditions such as pH, electrostatics, ionic strength, temperature, and local concentration is a valuable tool, because the aggregation processes can be understood in more detail (43–47). Modulation of electrostatic repulsion of Aβ has been shown to greatly influence the Aβ self-assembly mechanism by promoting surface-catalyzed secondary nucleation reactions (46, 47). Hence, in contrast to the divalent ions Zn(II) and Cu(II), the net charge of +1 makes Ag(I) ions a valuable comparative agent for studying the impact of electrostatics for metal ion modulation of peptide/protein aggregation.

In this project we used a combination of biophysical methods to study the characteristics of Aβ metal-ion binding to Ag(I) ions by characterizing the exchange dynamics and thermodynamics of the binding reaction and how this binding affects the fibrillization kinetics. Further, together with our previous results on Zn(II) (14), we rationalized a model for the determinants of modulation of Aβ self-assembly by transition metal ions. In short, 1) monomeric Aβ binds Ag(I) ions specifically in the N-terminal part, forming a dynamic metal-ion bound complex; 2) the weak metal-ion binding prevents monomeric Aβ from incorporation into fibrils; and 3) this leads to attenuation of the Aβ fibrillization kinetics in particular by reduction of the fibril-end elongation rate. Remarkably, these results are strikingly similar to effects of Zn(II) ions (14), which suggests a common mechanism of interaction of monovalent Ag(I) and divalent Zn(II) ions with Aβ peptides. Taken together, the metal-ion binding redirects Aβ monomers from fibril formation, retarding the overall Aβ fibrillization, in particular by reducing fibril-end elongation. This study hence links quantitatively the microscopic perturbation of metal-ion binding to Aβ monomers with its effect on the bulk peptide aggregation process.

## Results and discussion

### Ag(I) ions predominately retard Aβ fibril-end elongation

Fibrillization kinetics can be monitored using different fluorescent dyes that detect amyloid formation (48, 49). In this study, we simultaneously measured the aggregation kinetics of 20 μm Aβ_40_ and 5 μm Aβ_42_ at +37 °C under quiescent conditions using pentameric formyl thiophene acetic acid (pFTAA) (49) and thioflavin T (ThT) (48) (Figs. S1 and S2). With pFTAA we observed the typical sigmoidal aggregation kinetic traces (6, 50), whereas the monitoring of aggregation by ThT is interfered by interactions of Ag(I) and ThT (52, 53) (Fig. S1), which makes ThT unsuitable for aggregation kinetics in the presence of Ag(I) (supporting text).

**Figure 1.**
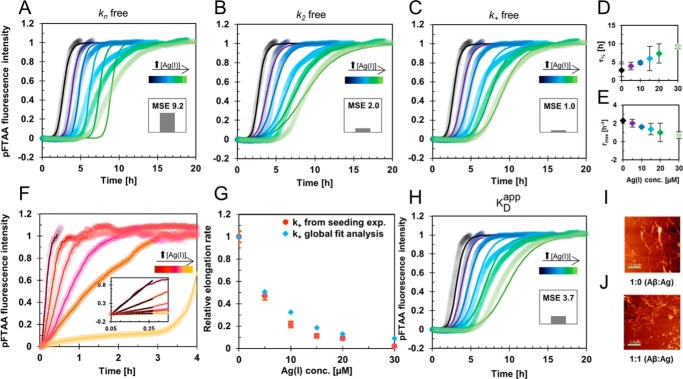
**Aβ fibrillization retardation in the presence of Ag(I) ions.** *A–C*, global fit analysis of aggregation traces of 20 μm monomeric Aβ_40_ incubated in the presence of 0–30 μm Ag(I) in 10 mm MOPS buffer, pH 7.2, at +37 °C under quiescent conditions. The global fits were constrained such that only one nucleation rate (*k*_n_, *k*_2_, or *k*_+_) is the single free fit parameter, revealing the best fit for *k*_+_ (normalized mean squared error (MSE), 1.0), followed by *k*_2_ (normalized MSE, 2.0) and *k*_n_ (normalized MSE, 9.2). *D* and *E*, parameters from sigmoidal curve fitting of the kinetic traces in *A–C*. The *error bars* represent standard deviation values from individual fits to four replicates (Fig. S1). *F*, elongation rates obtained from the initial slopes of highly seeded aggregation kinetics experiments, where 1 μm seeds were added to 20 μm monomeric Aβ_40_ with 0–30 μm Ag(I). The weighted average values calculated from four replicates are shown in *A–C* and *F. G*, relative *k*_+_ values obtained from the global fit analysis in *C* compared with values from seeding experiments in *F*, showing the same Ag(I) dependence of *k*_+_. *H*, global fit analysis applying a model, where the apparent free Aβ monomer concentration is determined by the dissociation constant *K*_*D*_^app^, revealing *K*_*D*_^app^ = 14.5 ± 0.2 μm (normalized MSE, 3.7). *I* and *J*, images from solid-state AFM of samples taken after fibrillization kinetic experiments showing similar Aβ_40_ fibril structures in the absence (*I*) and presence (*J*) of Ag(I) ions at 1:1 ratio. The *scale bar* represents 1 μm.

From pFTAA fluorescence experiments, we observed that the fibrillization kinetics of Aβ_40_ and Aβ_42_ are retarded by Ag(I) ions in a concentration-dependent manner (Fig. 1, *A–C*, and Fig. S1). We quantitatively analyzed the effect of Ag(I) ions on Aβ aggregation and found a clear Ag(I) concentration dependence of the aggregation halftime, τ_½_, and the maximum growth rate, *r*_max_ (Fig. 1, *D* and *E*), obtained from fitting a sigmoidal function to the aggregation trace. To obtain insights into the microscopic nucleation process of Aβ_40_, we analyzed the aggregation kinetics applying a global fit analysis using an integrated rate law (6, 50, 54, 55) (Fig. 1, *A–C*). The fibrillization process can selectively be differentiated into distinct nucleation events with their related microscopic rate constants, such as primary nucleation (*k*_n_), surface-catalyzed secondary nucleation (*k*_2_), and fibril-end elongation (*k*_+_) (5, 54, 55), and we assumed that similar microscopic nucleation events occur also in the presence of Ag(I).

To test the contribution of each microscopic rate constant, we globally fitted the kinetic curves with one single microscopic rate constant as an effective free fitting parameter by fixing the other two rate constants to constant values (Fig. 1, *A–C*). We found that the aggregation behavior could not be described with *k*_n_ as the sole fitting parameter. Although letting *k*_+_ be free better explains the observed aggregation data, using *k*_2_ as free parameter yields a better fit than for *k*_n_ but significantly worse than for *k*_+_ (Fig. 1 and Fig. S3).

To confirm that it is the elongation rate that is most affected by Ag(I) ions, we performed kinetics experiments in the presence of preformed seeds (Fig. 1*F* and Figs. S4 and S5). The initial slope of such kinetic trace is directly proportional to the elongation rate, and hence the isolated effect on *k*_+_ by silver ions can be estimated (5, 6, 14, 50). A high concentration of seeds was added to a monomeric peptide solution in the presence of different Ag(I) concentrations, and we found that the relative elongation rates obtained from these experiments agree very well with those from the global fit (Fig. 1*G*), providing further evidence that indeed the elongation rate is the rate constant mainly modulated by Ag(I). It is noteworthy that these findings do not exclude that there may be minor effects of Ag(I) on *k*_n_ and *k*_2_ as well.

Additionally, the experimental data were also fitted to a model, where the metal-bound population of Aβ monomers is assumed to be unavailable for fibril formation. The reduced aggregation-prone Aβ monomer pool can be described by an apparent free Aβ monomer concentration for each Ag(I) concentration. Similarly to a global fit analysis of aggregation kinetics with different Aβ concentrations (6, 50), the combined microscopic rate constants were globally fitted and constrained to the same values across all Ag(I) concentrations. In addition, an apparent dissociation constant *K*_*D*_^app^ was included as a global fit parameter, reflecting the apparently reduced free Aβ monomer concentration caused by the Ag(I) binding. This model describes reasonably well the observed aggregation kinetics, with *K*_*D*_^app^ = 14.5 ± 0.2 μm (Fig. 1*H*).

To investigate whether Ag(I) affects the final state of the Aβ_40_ fibrils, we used atomic force microscopy (AFM) and recorded images of the end-point samples from the aggregation experiments (Fig. 1, *I* and *J*, and Fig. S6). No detectable difference in the fibril morphology was found. This was further supported by circular dichroism (CD), which exhibit spectra showing similar β-structures of the aggregated state at all Ag(I) concentrations (Fig. S1, *G* and *H*).

We conclude that Ag(I) ions predominantly retard fibril-end elongation, whereas the structural state and the amount of the end-point fibrils are not affected (Fig. S1*C*). Hence, these metal ion interactions only modulate the fibrillization process, solely resulting in a delay of the aggregation process, whereas the aggregation mechanism and the final products are not altered.

### Interaction between Aβ monomers and Ag(I) ions

**Figure 2.**
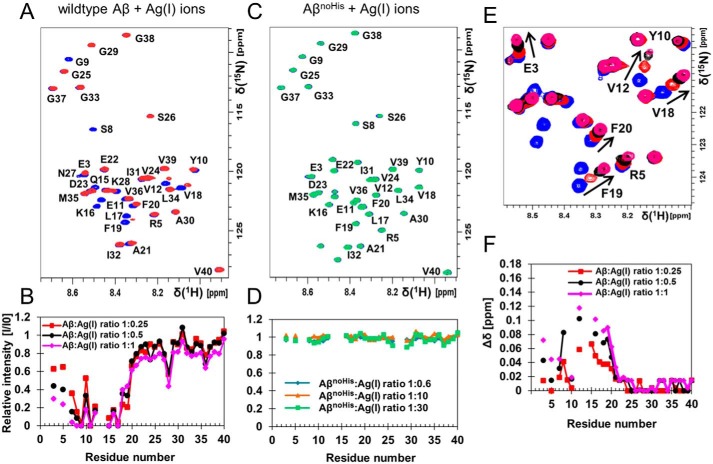
**Ag(I) ions bind specifically to the N-terminal part of monomeric Aβ peptide.** *A*, ^1^H-^15^N HSQC spectra of 80 μm monomeric ^13^C-^15^N-labeled Aβ_40_ peptides alone (*blue*) and with 20 μm Ag(I) ions, 4:1 Aβ_40_:Ag(I) (*red*) in 20 mm sodium phosphate buffer, pH 7.4, recorded at 278 K. *B*, relative intensities determined from the Ag(I) ion titration. *C* and *D*, ^1^H-^15^N HSQC spectra of ^15^N-labeled Aβ_40_ H6A,H13A,H14A mutant peptides (Aβ^noHis^) (*blue*) and in the presence of 1:30 Aβ_40_:Ag(I) ions (*green*), showing no changes in cross-peak intensities (*D*) and chemical shifts (*C*) in the presence of Ag(I) ions. *E*, magnification of the chemical shift changes observed in *A. F*, combined chemical shift changes from data in *A*. Hence, signal attenuation and chemical shift changes upon addition of Ag(I) are most prominent for the N-terminal residues.

Although aggregation experiments showed a reduction in the fibril-end elongation rate, these kinds of experiments do not reveal any details on the mechanism of metal-ion binding to monomeric peptide. To obtain high resolution information on the binding mechanism, we opted for NMR spectroscopy and recorded 2D ^1^H-^15^N HSQC and ^1^H-^13^C HSQC experiments (Fig. 2 and Fig. S7) at various silver ion concentrations. Addition of 20 μm Ag(I) ions to 80 μm ^13^C-^15^N-labeled Aβ_40_ (Ag(I):Aβ_40_ ratio of 1:4) resulted in an immediate attenuation of signal intensities of cross-peaks corresponding to N-terminal residues in both the ^15^N- (Fig. 2, *A* and *B*) and ^13^C-edited spectra (Fig. S7). In addition, induced chemical shift changes were observed (Fig. 2, *E* and *F*, and Fig. S7), indicating Ag(I)-induced conformational changes in the N terminus of Aβ. To exclude specific effects from Ag(I)–buffer interactions, we performed experiments in different buffers and found similar binding patterns (Fig. S8). The Ag(I) ion binding is reversible, as probed by an added chelator (1,10-phenanthroline) to a sample containing both Aβ and Ag(I) ions (Fig. S9). The loss of signal intensity and chemical shift differences induced by Ag(I) ions immediately returned to the original values in the presence of the chelator. SDS micelles constrain Aβ in a monomeric state while keeping the metal binding N terminus disordered in solution (56) and indeed while bound to SDS micelles similar binding pattern as in buffer solution was found (Fig. S10). Hence, we can conclude that Ag(I) ions bind to Aβ monomers (supporting text and Fig. S10). The ^1^H-^15^N HSQC cross-peak intensities of the histidine residues (His^6^, His^13^, and His^14^) in Aβ are only very weak under present conditions, whereas cross-peak intensities both in the aliphatic and in the aromatic region are detectable in the ^1^H-^13^C HSQC spectra, (Fig. S7). All histidines are clearly affected by the presence of Ag(I) ions and show ∼75–80% reduction of the initial signal intensity at 20 μm Ag(I). Neighboring residues to the histidines in the sequence such as Arg^5^, Ser^8^, Val^12^, and Gln^15^ are also affected, as well as Asp^1^, similar to the effect of Zn(II) ions (28).

To further verify that the histidines are the metal-binding ligands, we performed ^1^H-^15^N HSQC titration experiments on a histidine-free H6A,H13A,H14A-^15^N-Aβ_40_ variant (Aβ_40_^noHis^), and we found that the addition of more than 10-fold excess of Ag(I) ions neither caused any signal loss nor any chemical shift changes (Fig. 2, *C* and *D*). Moreover, 1D proton experiments for nonlabeled Aβ_40_ were conducted in D_2_O (Fig. S11*A*). Here the nonexchangeable imidazole proton signals are observed as three peaks ∼7.7 ppm, and upon titration of Ag(I) ions these signals were broadened. We conclude that the three histidines His^6^, His^13^, and His^14^ are involved in silver ion coordination.

The dissociation constant can be calculated from the signal loss in NMR HSQC data, assuming that the line broadening effect is linearly coupled to the bound population and is ∼3 μm in 20 mm sodium phosphate buffer, pH 7.4 (Table S2 and Fig. S12). Calculating the dissociation constant from induced chemical shifts instead gives consistent values (Table S2 and Fig. S12). In addition, intrinsic Tyr^10^ fluorescence experiments were conducted in which Ag(I) ions decrease the Tyr^10^ fluorescence intensity, and this phenomenon was used for a direct estimation of the dissociation constant (supporting text). The obtained values agree well with the values determined by NMR data and from analysis of the kinetics data (Fig. 1*H*). The values for the dissociation constant vary thus in a relatively narrow interval between 3 and 15 μm depending on the applied technique and experimental conditions (Table S2).

### Silver ions induce a more compact structure in Aβ

**Figure 3.**
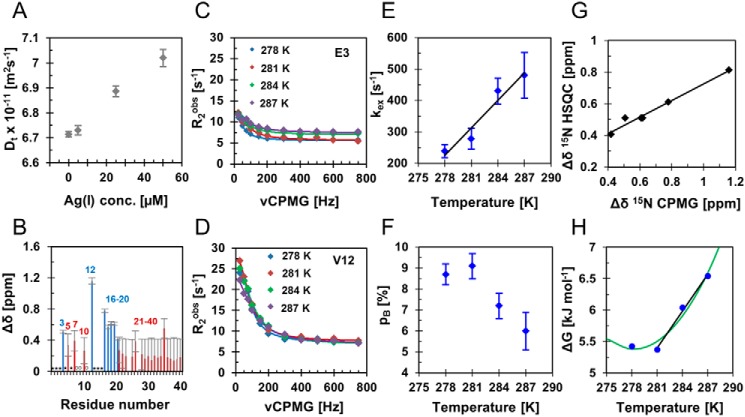
**Chemical exchange between free Aβ and an Aβ–Ag(I) ion complex.** *A*, translational diffusion coefficients of 80 μm monomeric Aβ_40_ in the absence and presence of 5–50 μm Ag(I) ions at 281 K. The average and standard deviation values were determined from five repeated measurements. *B–F*, relaxation dispersion was measured for 80 μm ^15^N-labeled Aβ_40_ with two different Ag(I) concentrations (4 and 6 μm). *B*, chemical shift differences from an individual data set with 6 μm Ag(I). *Blue bars* show the residues exhibiting significant chemical exchange (*p* < 0.01), which were included in the full analysis, whereas *red bars* correspond to residues with no significant chemical exchange. Residues marked with an *open circle* were not observed because of low signal intensity, and residues marked with an *asterisk* exhibited too fast exchange with the solvent or spectral overlap. *C* and *D*, relaxation dispersion profiles from two selected N-terminal residues at four different temperatures with 6 μm Ag(I). *E* and *F*, the temperature dependence of the global fit parameters, the chemical exchange rate, *k*_ex_, and bound population, *p*_B_. *G*, chemical shift changes from ^1^H-^15^N HSQC experiments plotted against the chemical shift differences from relaxation dispersion, displayed in *B*, revealing good correlation. *H*, Gibbs free energy for the four different temperatures shown for 6 μm Ag(I). At higher temperatures (≥281 K) the data points could be fitted linearly, whereas the whole data set exhibits a nonlinear dependence (*green*), described by an equation including a heat capacity difference (supporting text).

To examine whether the Ag(I) ion interaction causes the Aβ peptide to fold upon coordination of the metal ion, pulse field gradient diffusion experiments (57) were conducted at different Ag(I) concentrations. The translational diffusion coefficient for 80 μm Aβ_40_ peptide increases in a concentration-dependent manner from 6.7 to 7.0 × 10^−11^ m^2^/s upon increasing Ag(I) concentration from 0 to 50 μm (Fig. 3*A*). From the translational diffusion coefficient, the hydrodynamic radius (*R*_H_) can be calculated using the Stokes–Einstein equation (58, 59). The apparent hydrodynamic radius decreases from 17.0 Å for Aβ_40_ alone to 16.3 Å in the presence of 50 μm Ag(I) ions, suggesting a slightly more compact Aβ peptide structure once bound to the Ag(I) ion, without significant changes in the secondary structure content (Fig. S11*B*). Because the observed *R*_H_ is the population-weighted mean of the free and bound state, the hydrodynamic radius of the bound state can be estimated to be 16.0 Å, indicating a significant compactification upon metal-induced folding.

### Chemical exchange between Aβ–Ag(I) complex and free peptide

The loss of NMR signal of ^15^N-Aβ_40_ upon Ag(I) addition presumably originates from chemical exchange effects. Transient structures and dynamical features on the microsecond to millisecond time scale (typically underlying exchange broadening) are suitable to quantify using NMR relaxation dispersion experiments (60). We applied ^15^N Carr–Purcell–Meiboom–Gill (CPMG)–based pulse schemes (61–63) to characterize the influence of Ag(I) ions on 80 μm Aβ_40_ (Fig. 3, *B–F*), and we observed relaxation dispersion profiles in the presence of 4 and 6 μm Ag(I) at four different temperatures (278–287 K) (Figs. S13 and S14, and Table S3).

Especially, the N-terminal residues exhibit high-amplitude relaxation dispersion profiles, where seven residues show significant relaxation dispersion (F-test *p* value <0.01), and these residues were used for further analysis (Table S3). The seven residues are in close proximity to the histidines, the metal-binding ligands. Notably, also in the presence of SDS micelles, constraining Aβ as a monomer, the same N-terminal residues show relaxation dispersion profiles, confirming that the chemical exchange process can be attributed to Ag(I) binding to monomeric Aβ (supporting text and Figs. S10 and S15). In contrast, we have previously reported that Aβ_40_ peptides without metal ions do not show any relaxation dispersion profiles in this NMR time regime (14).

The relaxation dispersion profiles fit to a two-state exchange model (14, 64), which allows determination of the population of the free and bound states, (1 − *p*_B_) and *p*_B_, the chemical exchange rate, *k*_ex_, between the two states, the absolute value of chemical shift differences, |Δδ_N_|, and intrinsic transverse relaxation rate *R*_2_^0^ (Fig. 3, *E* and *F*, and Table S4). Although the latter two parameters, *R*_2_^0^ and |Δδ_N_|, are residue-specific parameters, the exchange rate and populations can be applied as global fitting parameters and constrained to the same values for all residues. We found that a model with a temperature-dependent *p*_B_ (referred to as model 1) best describes the data (Table S5). In this model, the exchange rate *k*_ex_ linearly increases and the population *p*_B_ decreases with increasing temperatures. Under the chosen experimental conditions, ∼6–9% of the Aβ population is bound to a silver ion at any given time point (Fig. 3*F*) and exchanges between the Ag(I)-bound and the free state with an exchange rate of 200 to 500 s^−1^ (Fig. 3*E*).

The chemical shift differences correlate well with the values obtained from the titration experiments monitored by ^1^H-^15^N HSQC experiments, indicating that the same structural state from the same process is observed (Fig. 3*G*). Interestingly, the exchange and population parameters for Ag(I) are in the same order as the ones previously determined for zinc ions (14). When calculating *K*_*D*_^app^ values from relaxation dispersion data, these values were estimated to ∼1 μm at 281 K (Table S4). This value is in the same order of magnitude compared with the *K*_*D*_^app^ values determined by HSQC titration (Table S2). To further confirm the model with a two-state exchange process, two different Ag(I) ion concentrations were compared. The exchange rate determined from the global fit at 6 μm Ag(I) ions does not significantly differ from the one at 4 μm Ag(I) ions and can be constrained to the same value in the fitting procedure (Tables S4 and S5). In contrast, the populated state (*p*_B_) does increase with ∼30% as expected for this increase in concentration (Table S4). This observation shows that the signal intensity loss and chemical shift changes in HSQC spectra upon Ag(I) ions titration are linearly coupled to the bound Aβ population. The *p*_B_ parameter is temperature-dependent and related to the Gibbs free energy difference, Δ*G*, between the free and the bound state via the equilibrium constant (supporting text and Fig. 3*H*). Notably, at a higher temperature (≥281 K), the data exhibit a linear temperature dependence, which reflects non–temperature-dependent contributions of the enthalpy and entropy. However, the whole data set fits best to temperature-dependent enthalpy and entropy terms, reflected in a heat capacity difference between the free and the bound state (supporting text). We found that the binding reaction is favored by enthalpy but disfavored by entropy. Together, the Gibbs free energy differences are small, yielding an unstable final fold at all temperatures (Fig. 3*H*, supporting text, and Tables S4 and S6).

### Insights into metal-ion binding mechanisms and effects on aggregation comparing Ag(I) and Zn(II)

The weak Ag(I) binding transiently removes Aβ monomers from the pool of aggregation-prone monomeric species, which are available to be incorporated into the fibrils (Figs. 1 and 3). Because the metal-ion binding is weak, eventually all Aβ peptides fibrillate. This analysis hence directly links the metal ion interactions with monomeric Aβ to the overall retardation effect of fibril formation.

**Figure 4.**
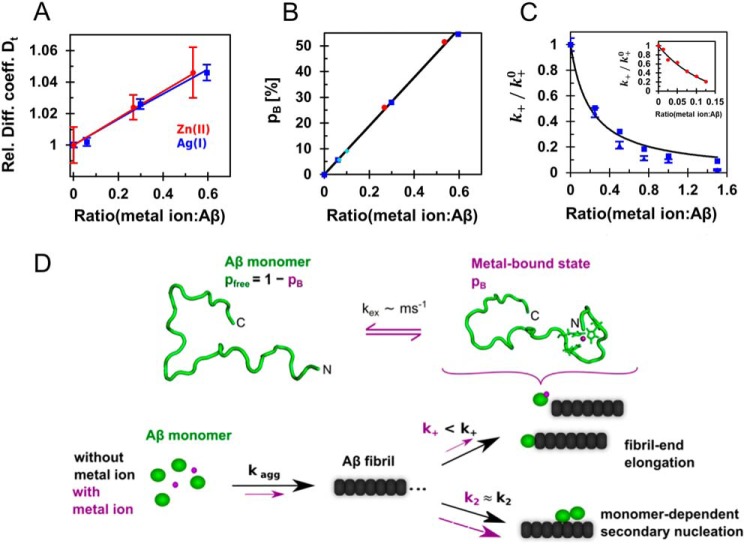
**Model for the role of Aβ:metal ion complexes in Aβ fibrillization using substoichiometric concentrations of Ag(I) and Zn(II) ions.** *A*, global fit of diffusion data where data from Ag(I) (*blue*) were fitted together with previously reported data for Zn(II) (14) (*red*) using a two-state model, revealing an increased relative diffusion coefficient (*Rel. Diff. coeff.*) of the bound/folded state by *D*_B_/*D*_free_ = 1.087 ± 0.002. *B*, the metal-bound populations, *p*_B_, are determined by the apparent dissociation constants for the respective metal ions and exhibit a linear dependence on the metal ion:Aβ ratio, here shown for the population corresponding to the diffusion data in *A*. The bound populations from relaxation dispersion experiments of Ag(I) (*turquoise*) confirm this relation. *C*, the relative elongation rates, *k*_+_, of Zn(II) (*red*) and Ag(I), from seeding (*blue triangles*) and global fit analysis (*blue squares*), are plotted against the metal ion:Aβ ratio, and fitted with Equation 5, revealing an apparent dissociation constant of *K*_*D*_^app^ = 4.1 ± 0.4 μm for Ag(I) ions and *K*_*D*_^app^ = 1.2 ± 0.2 μm for Zn(II) ions (*inset*). These values are similar as determined by other methods for Ag(I) (Table S2) and determined previously for Zn(II) (28). These findings indicate that Ag(I) and Zn(II) interact with monomeric Aβ in a remarkably similar manner. *D*, monomeric Aβ binds transition metal ions at the N terminus, forming a compact, histidine-coordinated fold. This metal–peptide complex is not stable and exchanges with the free peptide on the millisecond time scale. Aβ fibrils are formed through secondary nucleation mechanisms, in addition to fibril-end elongation, where the latter is predominantly attenuated by the presence of metal ions. Hence, these binding processes reduce the apparent available pool of free monomeric Aβ, resulting in a retardation of the overall fibrillization.

To distinguish whether this is solely a Ag(I) ion effect or whether it is a general metal-ion modulation effect of the Aβ fibrillization, we compared the results from monovalent Ag(I) with our previously reported findings from divalent Zn(II) (14) to be able to elucidate the impact of charge of the transition metal ion on the nucleation mechanism and binding characteristics. An induced folding of the N terminus upon metal-ion binding is a shared feature reflected in a decreased hydrodynamic radius of the Zn(II)–Aβ (14) and Ag(I)–Aβ complexes (Fig. 3*A* and Table S7). In fact, normalized diffusion data of Ag(I) and Zn(II) can be fitted together, constraining the normalized diffusion coefficient for the bound state, *D*_B_/*D*_free_, to the same values for both metal ions. This analysis revealed an increased value of *D*_B_/*D*_free_ = 1.087 ± 0.002, reflecting a decreased hydrodynamic radius by a factor 0.92 for the metal ion-bound/“folded” state (Fig. 4*A*). Interestingly, unlike in the presence of Zn(II), we observed significant chemical shift changes in ^1^H-^15^N HSQC resonances for Ag(I), indicating somewhat modulated exchange kinetics. Overall, both ions display similar binding regions with similar exchange dynamics; however, Zn(II) ions act at lower metal ion:Aβ ratios.

In fact, when plotting the population of the metal-bound states estimated by the *K_D_* values against the metal ion:Aβ ratio, a linear relation is evident (Fig. 4*B*). Notably, the bound population of Ag(I), as determined by relaxation dispersion experiments, agrees well with this prediction (Fig. 4*B*). For the bound/folded state, this suggests that the association mechanisms are very similar and determined by the respective dissociation constant. Comparing the thermodynamics for the binding, it turned out that both metal ions induce an enthalpy-favorable, yet unstable, final Aβ fold, where the heat capacity in the presence of Ag(I) is consistent with the more accurately determined value for Zn(II) (Table S7). Hence, these findings suggest strikingly similar binding mechanisms for both metal ions, where the lower binding efficiency for Ag(I) ions may stem from lower charge and larger ionic radius.

Specific interaction with monomeric Aβ resulting in reduction of the elongation rate is seemingly a common mechanism for the investigated metal ions. Indeed, the relative elongation rates for both Ag(I) and Zn(II) ions (14) can be fitted with a model for metal-ion binding to Aβ monomers as a function of an apparent binding constant, revealing *K*_*D*_^app^ = 4.1 ± 0.4 μm and 1.2 ± 0.2 μm for Ag(I) and Zn(II), respectively (Fig. 4*C*). These values are similar as obtained here by other methods for Ag(I) (Table S2) and reported previously for Zn(II) (28). This analysis hence suggests that the reduction of free Aβ monomer population by metal ion-bound state causes the decrease of the apparent fibril-end elongation rates.

### Concluding remarks

Taken together, we rationalized the mechanisms of action of transition metal-ion binding to Aβ in a schematic model (Fig. 4*D*). Metal-ion binding causes a N-terminal fold in Aβ with a histidine coordination of the metal ion, where the Aβ peptide exchanges at a millisecond time scale between the free and bound, folded states. This folded state is inert to fibril elongation. Hence, the pool of monomeric species available to be incorporated into the fibrils is reduced, retarding the overall Aβ fibrillization. Whereas also primary and secondary nucleation are dependent on the Aβ monomer concentration, the metal-ion interaction mainly affects the process of fibril-end elongation, because the elongation reaction involves a folding event when integrating Aβ into the fibril. This elongation event presumably includes multiple steps, where in a first step the peptide binds to the fibril end, followed by a folding event representing the conversion from a predominantly unstructured state to a β-structure conformation (66). In contrast, primary and secondary nucleation do not require a full β-structure formation to form the amyloid state. Hence the elongation event is particularly dependent on modulation of the folding thermodynamics, where the metal-bound state is unable to adopt a productive fold on the fibril end. However, because the metal-ion binding is weak, presumably much weaker than the affinity of the monomer to the fibrillar state, eventually almost all Aβ peptides are incorporated into the fibrils. This is also reflected when comparing the Gibbs free energy values of the fibril elongation event, which was determined to approximately −38 kJ/mol (67), and the value of the metal-bound state in the range of 5–7 kJ/mol.

To conclude, our analysis hence establishes the link between microscopic metal-ion interactions with monomeric Aβ and its macroscopic retardation effect of fibril formation, providing detailed mechanistic insights into modulation of Aβ self-assembly. We hence could further develop our previous model for Zn(II) (14) for both mono- and divalent metal ions and were able to show here in a strictly quantitative manner that the population of the aggregation-inert metal ion-bound state causes the retardation of Aβ fibrillization. These insights might be beneficial to interfere with specific Aβ nucleation events, which potentially prevents toxic pathways (68–70), and thereby find efficient ways for treatment of protein/peptide misfolding-related disorders.

## Experimental procedures

### Sample preparation

Recombinant Aβ peptides with the sequence ^1^DAEFRHDSGYEVHHQKLVFFAEDVGSNKGAIIGLMVGGVV^40^ (IA) were used in this study. Non-, ^15^N-, and ^13^C-^15^N-labeled Aβ_40_ peptides were bought lyophilized from AlexoTech AB, and nonlabeled Aβ_42_ peptides were purchased from rPeptide. Silver(I) acetate, silver(I) nitrate, and 1,10-phenanthroline were purchased from Sigma–Aldrich, and the Ag(I) ion concentration was determined by weight. For NMR experiments, the lyophilized peptides were dissolved in 10 mm NaOH, pH 12, at 1 mg ml^−1^; sonicated in an ice-water bath; and diluted to the desired concentration in the selected buffer. For kinetics experiments, the lyophilized peptides were dissolved in 6 m guanidium hydrochloric acid, pH 7.2, and prepared with size-exclusion chromatography using a Superdex 75 10/300 GL column from GE Healthcare to remove preformed aggregates (supporting text).

### pFTAA and ThT fibrillization kinetics using fluorescence spectroscopy

For aggregation kinetics experiments, 20 μm Aβ_40_ in 10 mm MOPS buffer, pH 7.2, was supplemented with 0.3 μm pentameric formyl thiophene acetic acid (pFTAA) (49) or 40 μm thioflavin T (ThT) (48, 71) and different concentrations of Ag(I) ions (0–30 μm). The fluorescence intensity was monitored over time with a FLUOstar Omega microplate reader from BMG LABTECH (Germany) each minute (for seeded samples) or each third minute in a 384-well plate, 30–40 μl/well, at +37 °C. Three or four replicates per condition were measured. Both raw data and weighted average kinetic traces were analyzed using sigmoidal curve fitting (72) (supporting text). For each type of kinetic experiments, at least three measurements were performed with qualitatively similar results.

#### 

##### Global fit analysis

Global fit analysis of the kinetic curves using the model presented by Meisl *et al.* (50) was performed where the Aβ fibrillization process under quiescent conditions is described as a monomer-dependent process with three different microscopic rate constants (6), *k*_n_ as the primary nucleation rate constant with reaction order *n*_c_ = 2, *k*_+_ as the elongation rate constant, and *k*_2_ as the secondary nucleation rate constant with the reaction order *n*_2_ = 2 (6, 50). The kinetic curves from 20 μm Aβ_40_ were globally fitted to a multistep secondary nucleation model with a Michaelis constant *K_M_* of 12.5 μm (14). Two of the rate constants *k*_+_, *k*_2_, and *k*_n_ were held constant, whereas the third rate constant was allowed to vary. The global fit analysis was performed with IgorPro 7 (WaveMetrics) and the Amylofit interface (73).

##### Seeding experiments

Seeding experiments were conducted with a fixed concentration of seeds of 1 μm supplemented to 20 μm monomeric Aβ_40_. The seeds were prepared from homogenized fibrillated samples by sonication, and the seed concentration was determined from the initial Aβ_40_ monomer concentration. The relative elongation rate constant, *k*_+_, was determined from the initial rate of the derived concave kinetic curves.

We applied a model where the kinetic traces at different Ag(I) concentrations are described by an apparent free Aβ monomer concentration, which is determined by an apparent dissociation constant, *K*_*D*_^app^. For a two-state exchange, *K*_*D*_^app^ can be described by Equation 1 based on the Ag(I)-bound population, *p*_B_, and the initial concentrations of Aβ monomers and Ag(I) ions (14).

(Eq. 1) $$K_{D}^{\text{app}}=\frac{\left(1-p_{\text{B}}\right)\cdot \left({\left[\text{Ag}\left(\text{I}\right)\right]}_{0}-p_{\text{B}}{\left[\text{A}\beta \right]}_{0}\right)}{p_{\text{B}}}$$

The apparent Aβ monomer concentration is then given by [Aβ]^app^ = [Aβ]_0_ (1 − *p*_B_), where *p*_B_ is a function of *K*_*D*_^app^, which can be derived from Equation 1. The aggregation traces were then globally fitted applying a secondary nucleation model (6), where *k*_n_*k*_+_, *k*_2_*k*_+_, and *K*_*D*_^app^ are global fit parameters, which were constrained to the same value across all values of [Aβ]^app^. Hence, all fitting parameters are globally constrained, and this approach facilitates testing the model of an apparent free Aβ monomer concentration that is determined by *K*_*D*_^app^.

### Solid-state AFM imaging and circular dichroism spectroscopy

Samples from the end of a fibrillization kinetic experiment were used for AFM imaging and CD measurements. CD spectra were also recorded for a titration series of Ag(I) ions onto monomeric Aβ_40_ in 20 mm sodium phosphate buffer, pH 7.4 (supporting text).

### NMR spectroscopy

Most NMR experiments were performed on a 700 MHz Bruker Avance spectrometer equipped with a cryogenic probe, if not stated differently. The 2D NMR ^1^H-^15^N HSQC experiments used 80 μm WT ^13^C-^15^N-Aβ_40_ or 80 μm ^15^N-labeled mutant Aβ_40_ (H6A,H13A,H14A) (referred to as Aβ_40_^noHis^) in 20 mm sodium phosphate buffer, pH 7.4 (90/10 H_2_O/D_2_O), and were performed at 278 K. The temperature was calibrated with an external thermometer. To investigate the Ag(I) ion-binding capacity to Aβ_40_ situated in a membrane-mimicking system, SDS micelles were used, applying 50 mm d-SDS (> critical micelle concentration) and 170 μm ^15^N-Aβ_40_. For the 2D spectra, the relative intensities for each amide cross-peak were determined from the amplitude of the cross-peaks. The combined chemical shift changes were calculated from Equation 2 (74, 75).

(Eq. 2) $$\Delta \delta ={\left(\left({\left(\frac{\Delta \delta_{\text{N}}}{5}\right)}^{2}+{\left(\Delta \delta_{\text{H}}\right)}^{2}\right)/2\right)}^{1/2}$$

The spectra were referenced to the ^1^H signal of trimethylsilylpropanoic acid. The Aβ_40_ amide cross-peak assignment in the HSQC spectra was performed by comparison with previously published work (24, 28, 56).

Pulse field gradient diffusion experiments were performed on a 600 MHz Bruker Avance spectrometer with 80 μm Aβ_40_ in 20 mm sodium phosphate buffer, pH 7.4, in D_2_O at 281 K with different Ag(I) concentrations. A standard sample of 1% H_2_O, 0.1% DSS and 0.1 mg ml^−1^ GdCl_3_ in D_2_O was used to calibrate the pulse field gradients. The translational diffusion coefficient (*D*_t_) was determined by one-component analysis, and the hydrodynamic radius (*r*_H_) was determined from the diffusion coefficient (76) using the Stokes–Einstein relation (58, 59). The diffusion data were further analyzed using a two-state model,

(Eq. 3) $$D_{\text{obs}}=p_{\text{free}}D_{\text{free}}+p_{\text{B}}D_{\text{B}}$$

in which *D*_free_ and *D*_B_ are the diffusion coefficient of the free and the bound state, respectively, with the respective populations. Additionally, a global fit analysis of the diffusion data from Ag(I) and Zn(II) (14) was performed on normalized diffusion data (Fig. 4*A*), where the coefficients *D*_free_ and *D*_B_ were constrained to the same value for Ag(I) and Zn(II), and the values of *p*_B_ were calculated by resolving Equation 1 using the respective apparent dissociation constants, here 1 μm for Zn(II) and 3.5 μm for Ag(I) (from HSQC analysis at low temperature) (28) (Table S2).

^15^N-CPMG relaxation dispersion experiments (61–63) were recorded on 80 μm ^15^N-Aβ_40_ peptides with 6 μm or 4 μm Ag(I), in 20 mm sodium phosphate or 10 mm HEPES buffer at pH 7.4. The Ag(I) concentration was adjusted and recalculated to the same signal attenuation upon Ag(I) addition based on the 2D NMR HSQC titration experiments. The experiment was repeated at four different temperatures (278–287 K) with 3 K intervals for each Ag(I) ion concentration. The ^15^N-CPMG relaxation dispersion experiments were performed as pseudo 3D experiments. 7 or 11 different CPMG frequencies were used as delays for the refocusing pulse with a mixing time, *T*_CP_, of 120 ms. The transverse relaxation rate (*R*_2_^obs^) values were determined from the signal ratios by *R*_2_^obs^ = 1/*T*_CP_·ln(*I*/*I*_0_). The significance of each relaxation dispersion profile was assessed based on an F-test (*p* value < 0.01). A global fit analysis using a two-state exchange model (14, 64) was performed by fitting the residues displaying significant relaxation dispersion profiles. The intrinsic transverse relaxation rates *R*_2_^0^ and the chemical shift changes |Δδ_N_| are residue-specific parameters, whereas the population *p*_B_ and the exchange rate *k*_ex_ were set to the same value for all residues. The chemical shift changes (|Δδ_N_|) were assumed to be independent of the temperature, ≥Δδ*_N_*(HSQC) and constrained to the same value for all temperatures in the global fit analysis. We applied two models: model 1, in which *p*_B_ is temperature-dependent, and model 2, in which *p*_B_ is temperature-independent. The models were assessed with an F-test and using the Akaike information criterion (Table S5), yielding model 1 as the preferred one. In model 1 the exchange rate for the two Ag(I) concentrations can be constrained to the same value (referred to as model 1b), resulting in the same quality of the fits (Table S5). From model 1 thermodynamic parameters for the binding and folding reaction were calculated (supporting text).

NMR data were processed with the Topspin version 3.2 software or NMRPipe. The diffusion data were analyzed using MatLab, and the ^15^N-CPMG relaxation dispersion data were analyzed and fitted using IgorPro 7 (WaveMetrics).

### Dissociation constant determination for Ag(I) binding

Apparent dissociation constants of the Aβ–Ag(I) complex were determined for different conditions with different techniques. Relative intensities and chemical shift changes from ^1^H-^15^N HSQC spectra were extracted and plotted against the Ag(I) ion concentration. The data were globally fitted to a model assuming one binding site (Equation 4) (65). Additionally, the apparent dissociation constant was determined from fluorescence spectroscopy data (supporting text). No buffer corrections were made.

(Eq. 4) $$I=I_{0}+\frac{I_{\infty }-I_{0}}{2\cdot \left[\text{A}\beta \right]}\cdot \left(K_{D}^{\text{app}}+\left[\text{Ag}\left(I\right)\right]+\left[\text{A}\beta \right]-\sqrt{{\left(K_{D}^{\text{app}}+\left[\text{Ag}\left(I\right)\right]+\left[\text{A}\beta \right]\right)}^{2}-4\cdot \left[\text{Ag}\left(I\right)\right]\cdot \left[\text{A}\beta \right]}\right)$$

where *I*_∞_ is the intensity upon saturation, *I*_0_ is the initial intensity without Ag(I) ions, and *K*_*D*_^app^ is the apparent dissociation constant. From NMR relaxation dispersion experiments, an apparent *K*_*D*_^app^ value was determined using the bound population *p*_B_.

The relative elongation rate constants in Fig. 4*C* were fitted using an equation that describes the effect of metal-ion binding to Aβ monomers on the elongation rate, in terms of an apparent dissociation constant (51).

(Eq. 5) $$k_{+}/{k^{0}}_{+}=1/\left(1+{\left[\text{Me}\right]}_{0}/K_{D}^{\text{app}}\right)$$

### Data availability

All supporting data are available from the corresponding author upon request.

## Author contributions

C. W., J. D., A. G., and A. A. conceptualization; C. W., J. D., and A. A. formal analysis; C. W. and A. A. investigation; C. W. and A. A. visualization; C. W., J. D., and A. A. methodology; C. W. and A. A. writing-original draft; C. W. and A. A. project administration; J. J., H. B., and S. W. resources; J. J., H. B., and S. W. validation; J. J., H. B., S. W., J. D., and A. G. writing-review and editing; J. D., A. G., and A. A. supervision; A. G. funding acquisition.

## Supplementary Material

Supporting Information

## Footnotes

2 The abbreviations used are:

Aβ: amyloid-β
pFTAA: pentameric formyl thiophene acetic acid
ThT: thioflavin T
CPMG: Carr–Purcell–Meiboom—Gill
AFM: atomic force microscopy
HSQC: heteronuclear single quantum coherence.

## References

[1] Dudev T., and Lim C. 2008) Metal binding affinity and selectivity in metalloproteins: insights from computational studies. Annu. Rev. Biophys. 37, 97–116 10.1146/annurev.biophys.37.032807.125811 18573074

[2] Glenner G. G., and Wong C. W. 1984) Alzheimer's disease: initial report of the purification and characterization of a novel cerebrovascular amyloid protein. Biochem. Biophys. Res. Commun. 120, 885–890 10.1016/S0006-291X(84)80190-4 6375662

[3] Haass C., and Selkoe D. J. 2007) Soluble protein oligomers in neurodegeneration: lessons from the Alzheimer's amyloid β-peptide. Nat. Rev. Mol. Cell Biol. 8, 101–112 10.1038/nrm2101 17245412

[4] Meisl G., Michaels T. C. T., Arosio P., Vendruscolo M., Dobson C. M., and Knowles T. P. J. 2019) Dynamics and control of peptide self-assembly and aggregation. Adv. Exp. Med. Biol. 1174, 1–33 10.1007/978-981-13-9791-2_1 31713195

[5] Cohen S. I., Vendruscolo M., Dobson C. M., and Knowles T. P. 2012) From macroscopic measurements to microscopic mechanisms of protein aggregation. J. Mol. Biol. 421, 160–171 10.1016/j.jmb.2012.02.031 22406275

[6] Cohen S. I., Linse S., Luheshi L. M., Hellstrand E., White D. A., Rajah L., Otzen D. E., Vendruscolo M., Dobson C. M., and Knowles T. P. 2013) Proliferation of amyloid-β42 aggregates occurs through a secondary nucleation mechanism. Proc. Natl. Acad. Sci. U.S.A. 110, 9758–9763 10.1073/pnas.1218402110 23703910 PMC3683769

[7] Knowles T. P., Vendruscolo M., and Dobson C. M. 2014) The amyloid state and its association with protein misfolding diseases. Nat. Rev. Mol. Cell Biol. 15, 384–396 10.1038/nrm3810 24854788

[8] Faller P., and Hureau C. 2009) Bioinorganic chemistry of copper and zinc ions coordinated to amyloid-β peptide. Dalt. Trans. 7, 1080–1094 10.1039/b813398k 19322475

[9] Weibull M. G. M., Simonsen S., Oksbjerg C. R., Tiwari M. K., and Hemmingsen L. 2019) Effects of Cu(II) on the aggregation of amyloid-β. J. Biol. Inorg. Chem. 24, 1197–1215 10.1007/s00775-019-01727-5 31602542

[10] Wärmländer S., Tiiman A., Abelein A., Luo J., Jarvet J., Söderberg K. L., Danielsson J., and Gräslund A. 2013) Biophysical studies of the amyloid β-peptide: interactions with metal ions and small molecules. Chembiochem. 14, 1692–1704 10.1002/cbic.201300262 23983094

[11] Hane F., and Leonenko Z. 2014) Effect of metals on kinetic pathways of amyloid-β aggregation. Biomolecules 4, 101–116 10.3390/biom4010101 24970207 PMC4030978

[12] Wallin C., Luo J., Jarvet J., Wärmländer S. K. T. S., and Gräslund A. 2017) The amyloid-β peptide in amyloid formation processes: interactions with blood proteins and naturally occurring metal ions. Isr. J. Chem. 57, 674–685 10.1002/ijch.201600105

[13] Bousejra-ElGarah F., Bijani C., Coppel Y., Faller P., and Hureau C. 2011) Iron(II) binding to amyloid-β, the Alzheimer's peptide. Inorg. Chem. 50, 9024–9030 10.1021/ic201233b 21800824

[14] Abelein A., Gräslund A., and Danielsson J. 2015) Zinc as chaperone-mimicking agent for retardation of amyloid β peptide fibril formation. Proc. Natl. Acad. Sci. U.S.A. 112, 5407–5412 10.1073/pnas.1421961112 25825723 PMC4418866

[15] Ayton S., Lei P., and Bush A. I. 2013) Metallostasis in Alzheimer's disease. Free Radic. Biol. Med. 62, 76–89 10.1016/j.freeradbiomed.2012.10.558 23142767

[16] Barnham K. J., and Bush A. I. 2008) Metals in Alzheimer's and Parkinson's diseases. Curr. Opin. Chem. Biol. 12, 222–228 10.1016/j.cbpa.2008.02.019 18342639

[17] Ayton S., Lei P., and Bush A. I. 2015) Biometals and their therapeutic implications in Alzheimer's disease. Neurotherapeutics 12, 109–120 10.1007/s13311-014-0312-z 25354461 PMC4322070

[18] Lovell M. A., Robertson J. D., Teesdale W. J., Campbell J. L., and Markesbery W. R. 1998) Copper, iron and zinc in Alzheimer's disease senile plaques. J. Neurol. Sci. 158, 47–52 10.1016/S0022-510X(98)00092-6 9667777

[19] Wallin C., Kulkarni Y. S., Abelein A., Jarvet J., Liao Q., Strodel B., Olsson L., Luo J., Abrahams J. P., Sholts S. B., Roos P. M., Kamerlin S. C., Gräslund A., and Wärmländer S. K. 2016) Characterization of Mn(II) ion binding to the amyloid-β peptide in Alzheimer's disease. J. Trace Elem. Med. Biol. 38, 183–193 10.1016/j.jtemb.2016.03.009 27085215

[20] Faller P. 2009) Copper and zinc binding to amyloid-β: coordination, dynamics, aggregation, reactivity and metal-ion transfer. ChemBioChem. 10, 2837–2845 10.1002/cbic.200900321 19877000

[21] Morante S., Minicozzi V., Stellato F., Comai M., Dalla Serra M., Potrich C., and Meyer-Klaucke W. 2008) Identifying the minimal copper- and zinc-binding site sequence in amyloid-β peptides. J. Biol. Chem. 283, 10784–10792 10.1074/jbc.M707109200 18234670

[22] Wallin C., Friedemann M., Sholts S. B., Noormägi A., Svantesson T., Jarvet J., Roos P. M., Palumaa P., Gräslund A., and Wärmländer S. K. T. S. 2019) Mercury and Alzheimer's disease: Hg(II) ions display specific binding to the amyloid-β peptide and hinder its fibrillization. Biomolecules 10, E44 31892131 10.3390/biom10010044 PMC7022868

[23] Wallin C., Sholts S. B., Österlund N., Luo J., Jarvet J., Roos P.M., Ilag L., Gräslund A., and Wärmländer S. K. T. S. 2017) Alzheimer's disease and cigarette smoke components: effects of nicotine, PAHs, and Cd(II), Cr(III), Pb(II), Pb(IV) ions on amyloid-β peptide aggregation. Sci. Rep. 7, 14423 10.1038/s41598-017-13759-5 29089568 PMC5663743

[24] Roche J., Shen Y., Lee J. H., Ying J., and Bax A. 2016) Monomeric Aβ1–40 and Aβ1–42 peptides in solution adopt very similar Ramachandran map distributions that closely resemble random coil. Biochemistry 55, 762–775 10.1021/acs.biochem.5b01259 26780756 PMC4750080

[25] Danielsson J., Jarvet J., Damberg P., and Gräslund A. 2005) The Alzheimer β-peptide shows temperature-dependent transitions between left-handed 3_1_-helix, β-strand and random coil secondary structures. FEBS J. 272, 3938–3949 10.1111/j.1742-4658.2005.04812.x 16045764

[26] Eisenberg D., and Jucker M. 2012) The amyloid state of proteins in human diseases. Cell 148, 1188–1203 10.1016/j.cell.2012.02.022 22424229 PMC3353745

[27] Morris K. L., and Serpell L. C. 2012) X-ray fibre diffraction studies of amyloid fibrils. Methods Mol. Biol. 849, 121–135 10.1007/978-1-61779-551-0_9 22528087

[28] Danielsson J., Pierattelli R., Banci L., and Gräslund A. 2007) High-resolution NMR studies of the zinc-binding site of the Alzheimer's amyloid β-peptide. FEBS J. 274, 46–59 10.1111/j.1742-4658.2006.05563.x 17222176

[29] Faller P., Hureau C., and La Penna G. 2014) Metal ions and intrinsically disordered proteins and peptides: from Cu/Zn amyloid-β to general principles. Acc. Chem. Res. 47, 2252–2259 10.1021/ar400293h 24871565

[30] Rezaei-Ghaleh N., Giller K., Becker S., and Zweckstetter M. 2011) Effect of zinc binding on β-amyloid structure and dynamics: implications for Aβ aggregation. Biophys. J. 101, 1202–1211 10.1016/j.bpj.2011.06.062 21889458 PMC3164126

[31] Faller P., Hureau C., and Berthoumieu O. 2013) Role of metal ions in the self-assembly of the Alzheimer's amyloid-β peptide. Inorg. Chem. 52, 12193–12206 10.1021/ic4003059 23607830

[32] Tõugu V., Karafin A., Zovo K., Chung R. S., Howells C., West A. K., and Palumaa P. 2009) Zn(II)- and Cu(II)-induced non-fibrillar aggregates of amyloid-β (1–42) peptide are transformed to amyloid fibrils, both spontaneously and under the influence of metal chelators. J. Neurochem. 110, 1784–1795 10.1111/j.1471-4159.2009.06269.x 19619132

[33] Pedersen J. T., Teilum K., Heegaard N. H., Østergaard J., Adolph H. W., and Hemmingsen L. 2011) Rapid formation of a preoligomeric peptide–metal–peptide complex following copper(II) binding to amyloid β peptides. Angew. Chem. Int. Ed. Engl. 50, 2532–2535 10.1002/anie.201006335 21370331

[34] Somavarapu A. K., Shen F., Teilum K., Zhang J., Mossin S., Thulstrup P. W., Bjerrum M. J., Tiwari M. K., Szunyogh D., Søtofte P. M., Kepp K. P., and Hemmingsen L. 2017) The pathogenic A2V mutant exhibits distinct aggregation kinetics, metal site structure, and metal exchange of the Cu^2+^–Aβ complex. Chemistry 23, 13591–13595 10.1002/chem.201703440 28815875

[35] Merril C. R., Goldman D., Sedman S.A., and Ebert M. H. 1981) Ultrasensitive stain for proteins in polyacrylamide gels shows regional variation in cerebrospinal fluid proteins. Science 211, 1437–1438 10.1126/science.6162199 6162199

[36] Chevallet M., Luche S., and Rabilloud T. 2006) Silver staining of proteins in polyacrylamide gels. Nat. Protoc. 1, 1852–1858 10.1038/nprot.2006.288 17487168 PMC1971133

[37] Eckhardt S., Brunetto P. S., Gagnon J., Priebe M., Giese B., and Fromm K. M. 2013) Nanobio silver: its interactions with peptides and bacteria, and its uses in medicine. Chem. Rev. 113, 4708–4754 10.1021/cr300288v 23488929

[38] Veronesi G., Gallon T., Deniaud A., Boff B., Gateau C., Lebrun C., Vidaud C., Rollin-Genetet F., Carrière M., Kieffer I., Mintz E., Delangle P., and Michaud-Soret I. 2015) XAS investigation of silver(I) coordination in copper(I) biological binding sites. Inorg. Chem. 54, 11688–11696 10.1021/acs.inorgchem.5b01658 26632864

[39] Grosas A. B., Kalimuthu P., Smith A. C., Williams P. A., Millar T. J., Bernhardt P. V., and Jones C. E. 2014) The tachykinin peptide neurokinin B binds copper(I) and silver(I) and undergoes quasi-reversible electrochemistry: towards a new function for the peptide in the brain. Neurochem. Int. 70, 1–9 10.1016/j.neuint.2014.03.002 24650723

[40] De Ricco R., Potocki S., Kozlowski H., and Valensin D. 2014) NMR investigations of metal interactions with unstructured soluble protein domains. Coord. Chem. Rev. 269, 1–12 10.1016/j.ccr.2014.02.014

[41] Valensin D., Padula E. M., Hecel A., Luczkowski M., and Kozlowski H. 2016) Specific binding modes of Cu(I) and Ag(I) with neurotoxic domain of the human prion protein. J. Inorg. Biochem. 155, 26–35 10.1016/j.jinorgbio.2015.11.015 26606290

[42] De Gregorio G., Biasotto F., Hecel A., Luczkowski M., Kozlowski H., and Valensin D. 2019) Structural analysis of copper(I) interaction with amyloid β peptide. J. Inorg. Biochem. 195, 31–38 10.1016/j.jinorgbio.2019.03.006 30884319

[43] Meisl G., Yang X., Frohm B., Knowles T. P., and Linse S. 2016) Quantitative analysis of intrinsic and extrinsic factors in the aggregation mechanism of Alzheimer-associated Aβ-peptide. Sci. Rep. 6, 18728 10.1038/srep18728 26758487 PMC4725935

[44] Yang X., Meisl G., Frohm B., Thulin E., Knowles T. P. J., and Linse S. 2018) On the role of sidechain size and charge in the aggregation of A β 42 with familial mutations. Proc. Natl. Acad. Sci. U.S.A. 115, E5849–E5858 10.1073/pnas.1803539115 29895690 PMC6042101

[45] Cohen S. I. A., Cukalevski R., Michaels T. C. T., Šarić A., Törnquist M., Vendruscolo M., Dobson C. M., Buell A. K., Knowles T. P. J., and Linse S. 2018) Distinct thermodynamic signatures of oligomer generation in the aggregation of the amyloid-β peptide. Nat. Chem. 10, 523–531 10.1038/s41557-018-0023-x 29581486 PMC5911155

[46] Abelein A., Jarvet J., Barth A., Gräslund A., and Danielsson J. 2016) Ionic strength modulation of the free energy landscape of Aβ40 peptide fibril formation. J. Am. Chem. Soc. 138, 6893–6902 10.1021/jacs.6b04511 27171340

[47] Meisl G., Yang X., Dobson C. M., Linse S., and Knowles T. P. J. 2017) Modulation of electrostatic interactions to reveal a reaction network unifying the aggregation behaviour of the Aβ42 peptide and its variants. Chem. Sci. 8, 4352–4362 10.1039/C7SC00215G 28979758 PMC5580342

[48] Biancalana M., and Koide S. 2010) Molecular mechanism of thioflavin-T binding to amyloid fibrils. Biochim. Biophys. Acta 1804, 1405–1412 10.1016/j.bbapap.2010.04.001 20399286 PMC2880406

[49] Klingstedt T., Aslund A., Simon R. A., Johansson L. B., Mason J. J., Nyström S., Hammarström P., and Nilsson K. P. 2011) Synthesis of a library of oligothiophenes and their utilization as fluorescent ligands for spectral assignment of protein aggregates. Org. Biomol. Chem. 9, 8356–8370 10.1039/c1ob05637a 22051883 PMC3326384

[50] Meisl G., Yang X., Hellstrand E., Frohm B., Kirkegaard J. B., Cohen S. I., Dobson C. M., Linse S., and Knowles T. P. 2014) Differences in nucleation behavior underlie the contrasting aggregation kinetics of the Aβ40 and Aβ42 peptides. Proc. Natl. Acad. Sci. U.S.A. 111, 9384–9389 10.1073/pnas.1401564111 24938782 PMC4084462

[51] Arosio P., Michaels T. C., Linse S., Månsson C., Emanuelsson C., Presto J., Johansson J., Vendruscolo M., Dobson C. M., and Knowles T. P. 2016) Kinetic analysis reveals the diversity of microscopic mechanisms through which molecular chaperones suppress amyloid formation. Nat. Commun. 7, 10948 10.1038/ncomms10948 27009901 PMC4820785

[52] Wang Y., Geng F., Xu H., Qu P., Zhou X., and Xu M. 2012) A label-free oligonucleotide based thioflavin-t fluorescent switch for Ag^+^ detection with low background emission. J. Fluoresc. 22, 925–929 10.1007/s10895-011-1031-z 22234459

[53] Makarava N., Parfenov A., and Baskakov I. V. 2005) Water-soluble hybrid nanoclusters with extra bright and photostable emissions: a new tool for biological imaging. Biophys. J. 89, 572–580 10.1529/biophysj.104.049627 15833997 PMC1366557

[54] Knowles T. P., Waudby C. A., Devlin G. L., Cohen S. I., Aguzzi A., Vendruscolo M., Terentjev E. M., Welland M. E., and Dobson C. M. 2009) An analytical solution to the kinetics of breakable filament assembly. Science 326, 1533–1537 10.1126/science.1178250 20007899

[55] Cohen S. I. A., Vendruscolo M., Welland M. E., Dobson C. M., Terentjev E. M., and Knowles T. P. J. 2011) Nucleated polymerization with secondary pathways: I. Time evolution of the principal moments. J. Chem. Phys. 135, 065105 10.1063/1.3608916 21842954 PMC5017532

[56] Jarvet J., Danielsson J., Damberg P., Oleszczuk M., and Gräslund A. 2007) Positioning of the Alzheimer Aβ(1–40) peptide in SDS micelles using NMR and paramagnetic probes. J. Biomol. NMR 39, 63–72 10.1007/s10858-007-9176-4 17657567

[57] Stejskal E. O., and Tanner J. E. 1965) Spin diffusion measurements: spin echoes in the presence of a time-dependent field gradient. J. Chem. Phys. 42, 288–292 10.1063/1.1695690

[58] Schultz S. G., and Solomon A. K. 1961) Determination of the effective hydrodynamic radii of small molecules by viscometry. J. Gen. Physiol. 44, 1189–1199 10.1085/jgp.44.6.1189 13748878 PMC2195139

[59] Achuthan S., Chung B. J., Ghosh P., Rangachari V., and Vaidya A. 2011) A modified Stokes–Einstein equation for Aβ aggregation. BMC Bioinformatics 12, (Suppl. 10) S13 10.1186/1471-2105-12-S10-S13 PMC3236835 22166081

[60] Palmer A. G. 3rd, Kroenke C. D., and Loria J. P. 2001) Nuclear magnetic resonance methods for quantifying microsecond-to-millisecond motions in biological macromolecules. Methods Enzymol. 339, 204–238 10.1016/S0076-6879(01)39315-1 11462813

[61] Carr H. Y., and Purcell E. M. 1954) Effects of diffusion on free precession in nuclear magnetic resonance experiments. Phys. Rev. 94, 630–638 10.1103/PhysRev.94.630

[62] Meiboom S., and Gill D. 1958) Modified spin-echo method for measuring nuclear relaxation times. Rev. Sci. Instrum. 29, 688–691 10.1063/1.1716296

[63] Tollinger M., Skrynnikov N. R., Mulder F. A., Forman-Kay J. D., and Kay L. E. 2001) Slow dynamics in folded and unfolded states of an SH3 domain. J. Am. Chem. Soc. 123, 11341–11352 10.1021/ja011300z 11707108

[64] Abelein A., Lang L., Lendel C., Gräslund A., and Danielsson J. 2012) Transient small molecule interactions kinetically modulate amyloid β peptide self-assembly. FEBS Lett. 586, 3991–3995; Correction (2013) *FEBS Lett*. **587**, 1452 10.1016/j.febslet.2012.09.035 23058290

[65] Tiiman A., Luo J., Wallin C., Olsson L., Lindgren J., Jarvet J., Per R., Sholts S. B., Rahimipour S., Abrahams J. P., Karlström A. E., Gräslund A., and Wärmländer S. K. 2016) Specific binding of Cu(II) ions to amyloid-β peptides bound to aggregation-inhibiting molecules or SDS micelles creates complexes that generate radical oxygen species. J. Alzheimers Dis. 54, 971–982 10.3233/JAD-160427 27567855

[66] Cannon M. J., Williams A. D., Wetzel R., and Myszka D. G. 2004) Kinetic analysis of β-amyloid fibril elongation. Anal. Biochem. 328, 67–75 10.1016/j.ab.2004.01.014 15081909

[67] O'Nuallain B., Shivaprasad S., Kheterpal I., and Wetzel R. 2005) Thermodynamics of Aβ(1–40) amyloid fibril elongation. Biochemistry. 44, 12709–12718 10.1021/bi050927h 16171385

[68] Arosio P., Vendruscolo M., Dobson C. M., and Knowles T. P. 2014) Chemical kinetics for drug discovery to combat protein aggregation diseases. Trends Pharmacol. Sci. 35, 127–135 10.1016/j.tips.2013.12.005 24560688

[69] Cohen S. I. A., Arosio P., Presto J., Kurudenkandy F. R., Biverstal H., Dolfe L., Dunning C., Yang X., Frohm B., Vendruscolo M., Johansson J., Dobson C. M., Fisahn A., Knowles T. P. J., and Linse S. 2015) A molecular chaperone breaks the catalytic cycle that generates toxic Aβ oligomers. Nat. Struct. Mol. Biol. 22, 207–213 10.1038/nsmb.2971 25686087 PMC4595974

[70] Chen G., Abelein A., Nilsson H. E., Leppert A., Andrade-Talavera Y., Tambaro S., Hemmingsson L., Roshan F., Landreh M., Biverstål H., Koeck P. J. B., Presto J., Hebert H., Fisahn A., and Johansson J. 2017) Bri2 BRICHOS client specificity and chaperone activity are governed by assembly state. Nat. Commun. 8, 2081 10.1038/s41467-017-02056-4 29234026 PMC5727130

[71] Xue C., Lin T. Y., Chang D., and Guo Z. 2017) Thioflavin T as an amyloid dye: fibril quantification, optimal concentration and effect on aggregation. R. Soc. Open Sci. 4, 160696 10.1098/rsos.160696 28280572 PMC5319338

[72] Hellstrand E., Boland B., Walsh D. M., and Linse S. 2010) Amyloid β-protein aggregation produces highly reproducible kinetic data and occurs by a two-phase process. ACS Chem. Neurosci. 1, 13–18 10.1021/cn900015v 22778803 PMC3368626

[73] Meisl G., Kirkegaard J. B., Arosio P., Michaels T. C., Vendruscolo M., Dobson C. M., Linse S., and Knowles T. P. 2016) Molecular mechanisms of protein aggregation from global fitting of kinetic models. Nat. Protoc. 11, 252–272 10.1038/nprot.2016.010 26741409

[74] Williamson M. P. 2013) Using chemical shift perturbation to characterise ligand binding. Prog. Nucl. Magn. Reson. Spectrosc. 73, 1–16 10.1016/j.pnmrs.2013.02.001 23962882

[75] Lindgren J., Wahlström A., Danielsson J., Markova N., Ekblad C., Gräslund A., Abrahmsén L., Karlström A. E., and Wärmländer S. K. 2010) N-terminal engineering of amyloid-β-binding Affibody molecules yields improved chemical synthesis and higher binding affinity. Protein Sci. 19, 2319–2329 10.1002/pro.511 20886513 PMC3009399

[76] Cho C. H., Urquidi J., Singh S., and Robinson G. W. 1999) Thermal offset viscosities of liquid H_2_O, D_2_O, and T_2_O. J. Phys. Chem. B. 103, 1991–1994 10.1021/jp9842953

