# Supplementary material for: Metal ion coordination delays amyloid-β peptide self-assembly by forming an aggregation–inert complex
Source: J Biol Chem. 2020 Apr 2;295(21):7224–34. doi: 10.1074/jbc.RA120.012738 (PMC7247290; doi:10.1074/jbc.RA120.012738)
Supplement: Supporting Information [file supp_295_21_7224__index.html]

Metal ion coordination delays amyloid-β peptide self-assembly by forming an aggregation-inert complex — Mechanistic insights into Aβ self-assembly by metal ions — Metal ion coordination delays amyloid-β peptide self-assembly by forming an aggregation–inert complex — Mechanistic insights into Aβ self-assembly by metal ions — Supporting Information 

# Metal ion coordination delays amyloid-β peptide self-assembly by forming an aggregation–inert complex

## Supporting Information

- Supporting Information (to be published online) - Supporting Information including SI text, SI Figures and SI Tables
