# Supplementary material for: Metal ion coordination delays amyloid-β peptide self-assembly by forming an aggregation–inert complex
Source: J Biol Chem. 2020 Apr 2;295(21):7224–34. doi: 10.1074/jbc.RA120.012738 (PMC7247290; doi:10.1074/jbc.RA120.012738)
Supplement: Supporting Information [file supp_RA120.012738_158359_3_supp_504169_q85rnl.pdf]

# SUPPORTING INFORMATION

# Metal ion coordination delays amyloid-β peptide self-assembly by forming an aggregation-inert complex

## *Mechanistic insights into Aβ self-assembly by metal ions*

Cecilia Wallin[1], Jüri Jarvet[1], Henrik Biverstål[2,3], Sebastian Wärmländer[1], Jens Danielsson[1], Astrid Gräslund[1], Axel Abelein[2]*

[1] Department of Biochemistry and Biophysics, The Arrhenius Laboratories, Stockholm University, 106 91, Stockholm, Sweden

[2] Department of Neurobiology, Care Sciences and Society, Center for Alzheimer Research, Division of Neurogeriatrics, Karolinska Institutet, 141 52, Huddinge, Sweden

[3] Department of Physical Organic Chemistry, Latvian Institute of Organic Synthesis, Riga LV-1006, Latvia

*Corresponding author: Axel Abelein
Corresponding author e-mail address: axel.abelein@ki.se

# Table of Contents

**Supporting description** ……………………………………………………………………………………………

*Extended materials and methods*
*Fibrillization kinetics monitored by pFTAA*
*Interaction studies using CD*
*Thermodynamics of the Ag(I)-binding reaction*
*Transient binding of Ag(I) to monomeric Aβ stabilized by SDS micelles*

**Supporting Figures** ……………………………………………………………………………………….....................

*Figure S1. Comparison pFTAA and ThT as amyloid-reporting dyes for $Aβ_{40}$ and $Aβ_{42}$ fibrillization kinetics in the presence of Ag(I) ions*

*Figure S2. Control experiments with different concentrations of pFTAA*

*Figure S3. Residuals from the global fit using 20 µM $Aβ_{40}$ peptides incubated with Ag(I) ions (0, 5, 10, 15, 20, 30 µM Ag(I) ions) presented in Fig. 1 in the main manuscript*

*Figure S4. Raw data from seeding experiments shown in Fig. 1 in the main manuscript*

*Figure S5. Secondary nucleation is the dominant process for $Aβ_{40}$ peptide fibrillization in the presence of Ag(I) ions*

*Figure S6. Aβ fibrils formed in the absence and presence of Ag(I) ions indicate no obvious differences in fibril structure*

*Figure S7. Ag(I) ions bind specifically to the N-terminal part of the monomeric Aβ peptide inducing structural changes monitored by $^{1}H$-$^{13}C$-HSQC experiments*

*Figure S8. Ag(I) ions bind specifically to the N-terminal part of the monomeric Aβ peptide in HEPES, MOPS and sodium phosphate buffer at physiological pH and at pH 8*

*Figure S9. Reversible binding of Ag(I) ions to the N-terminal part of the monomeric Aβ peptide probed with the 1,10-Phenanthroline chelator*

*Figure S10. 2D NMR HSQC and $^{15}N$-CPMG relaxation dispersion experiments, chemical exchange between free Aβ peptide and an Aβ-Ag(I) complex when the Aβ peptides are bound to SDS micelles*

*Figure S11. Ag(I) ions bind specifically to histidine residues in the N-terminal part of monomeric Aβ peptide inducing local structural changes*

*Figure S12. Representative Ag(I) ion titration curves used for apparent dissociation constant $K_D^{app}$ determination presented in Table S2*

*Figure S13. $^{15}N$-CPMG relaxation dispersion profiles for all observable crosspeaks, 4 µM Ag(I) ions*

*Figure S14. $^{15}N$-CPMG relaxation dispersion profiles for all observable crosspeaks, 6 µM Ag(I) ions*

*Figure S15. $^{15}N$-CPMG relaxation dispersion profiles for all observable crosspeaks in SDS*

**Supporting Tables** ……………………………………………………………………………………….....................

*Table S1. Fundamental properties of silver-, zinc- and copper ions related to metal-protein coordination*

*Table S2. Apparent dissociation constants ($K_D^{app}$) determined from Ag(I) ions titration experiments using Y10 intrinsic fluorescence effects or signal attenuation/chemical shift changes in 1D or 2D NMR $^{1}H$-$^{15}N$-HSQC experiments*

*Table S3. Global fit parameters from the $^{15}N$-CPMG relaxation dispersion data*

*Table S4. Global fit parameters from $^{15}N$-CPMG relaxation dispersion data shown in the main manuscript in Fig. 3, and calculated dissociation constants ($K_D^{app}$) using Eq. 1 in the main manuscript and Gibbs free energy values ($ΔG_{U\text{-->}F}$).*

*Table S5. Global fit of $^{15}$N-CPMG relaxation dispersion data shown in the main manuscript in Fig. 3 with different constraints*

*Table S6. Thermodynamic parameters for Ag(I)-induced folding of the N-terminus of Aβ$_{40}$*

*Table S7. Biophysical Ag(I)-Aβ and Zn(II)-Aβ data comparison*

**References ……………………………………………………………………………………………………….**

# Supporting description

## EXTENDED MATERIALS AND METHODS

### Detailed sample preparation for kinetics experiments

For kinetics experiments, the lyophilized A$\beta_{40}$ and A$\beta_{42}$ peptides were dissolved in 6 M Guanidium hydrochloric acid pH 7.2 and prepared with size exclusion chromatography (SEC) using a Superdex 75 10/300 GL column from GE Healthcare to remove pre-formed aggregates. 2 mg ml$^{-1}$ A$\beta_{40}$ or A$\beta_{42}$ was injected to the equilibrated SEC column and eluted with a flow rate of 0.5 ml min$^{-1}$ in 10 mM MOPS buffer pH 7.2 at room temperature. The monomer peak was collected and the peptide concentration was determined by absorbance at 280 nm with an extinction coefficient of 1490 M$^{-1}$ cm$^{-1}$. All samples were kept on ice.

### pFTAA and ThT fibrillization kinetics using fluorescence spectroscopy

*Control experiments* using different concentrations (0-10 µM) of pentameric formyl thiophene acetic acid (pFTAA) in the absence and presence of 8 µM A$\beta_{42}$ peptides in 10 mM MOPS buffer pH 7.8 were performed. Eight replicates per conditions were measured. Excitation and emission filters were 485 nm and 520 nm, respectively.

The chosen concentrations of Thioflavin T (ThT) were motivated by prior studies for optimal fluorescence detection(1, 2). pFTAA has not been studied to the same extent as ThT, but relative lower concentrations than for ThT have been used in earlier studies(3) and higher concentrations have been reported to influence the properties of fibril toxicity(4). The pFTAA concentration of 0.3 µM was sufficiently high to report on amyloid formation without disturbing the system.

*Sigmoidal curve fitting* of aggregation traces was performed using Eq.S1 (5), where the aggregation halftime, $\tau_{½}$, and the maximum growth rate, $r_{max}$, can be determined.

$$\mathbf{F(t) = F_0 + \frac{A}{1+\exp\left[r_{max}(\tau_{½}-t)\right]}} \qquad \text{(Eq. S1)}$$

where $F_0$ is the fluorescence signal intensity baseline and A is the fluorescence intensity amplitude.

### Solid-state AFM imaging

Samples from the end of a fibrillization kinetic experiment were used for atomic force microscopy (AFM) imaging. The samples were diluted twice in Milli-Q water and put on freshly cleaved mica surfaces for 20 min incubation. Thereafter the mica surfaces were washed three times with Milli-Q water. The AFM imaging was performed on a Scan Asyst from Bruker Corporation operating in tapping mode in air, recording 5 x 5 µm topographical images at a resolution of 512-512 pixels.

### Circular dichroism spectroscopy

We conducted CD measurements on a Chirascan CD spectrometer (Applied Photophysics). The peptide secondary structures were studied by recording CD spectra under quiescent conditions at room temperature in the spectral range of 190-260 nm with a bandwidth of 1 nm, resolution/step size of 1 nm, and a time-per-point of 2 s. The background signal of water was subtracted, and the data was presented as mean residue molar ellipticity. The data was processed with a smoothing function of ten points.

A quartz cuvette with a 4 mm pathlength was used for 10 µM monomeric A$\beta_{40}$ in 20 mM sodium phosphate buffer pH 7.4 for a titration series of Ag(I) ions (1, 2, 3, 5, 10, 20, 50, 100, 500, 1000 µM). The titration was repeated two times. Sodium phosphate buffer was used since its absorption is suitable for recording the CD spectra in the chosen range.

A$\beta_{40}$ samples supplemented with pFTAA or ThT were taken from the end of the fibrillization kinetic experiments (initially 20 µM monomeric A$\beta_{40}$ peptides in 10 mM MOPS buffer pH 7.2). Wells within the same condition were pooled together and transferred to a 1 mm pathlength cuvette. A spectral range of 205-255 nm is shown for the samples because of the MOPS buffer absorption properties in the lower region of the spectral range.

### Dissociation constant determination for Ag(I) binding by intrinsic Tyr10 fluorescence quenching

Ag(I) ions decrease the Tyr10 fluorescence intensity and this phenomenon was used for a direct determination of the dissociation constant. The mechanism behind the decrease of Tyr10 fluorescence intensity upon Ag(I) ion titration is not yet understood in detail, but structural rearrangements upon Ag(I)-binding or water replacement might contribute to the fluorescence intensity reduction. Notably, the dissociation constants are very similar to the values obtained by NMR (Table S2).

For Tyr10 fluorescence experiments of 10 µM A$\beta_{40}$, a Jobin Yvon Horiba Fluorolog 3 was used with an excitation wavelength of 276 nm to record emission fluorescence spectra in the range of 290-350 nm at room temperature. The relative intensity was plotted and fitted to Eq. 4 (6) in the main manuscript. No buffer corrections were made.

### Two-state reaction model

A two-state model was applied to describe the Ag(I) binding and folding event:

$$A\beta + Ag^{+} \rightleftharpoons A\beta{:}Ag^{+}$$

where the Ag(I) binding is defined by the dissociation constant (Table S2). The Gibbs free energy and equilibrium constant of this reaction are given by Eq. S2 and S3.

$$\mathbf{K_{eq}^{U\to F} = p_B/p_{free} = p_B/(1 - p_B)} \quad \text{(Eq. S2)}$$

$$\boldsymbol{\Delta G_{U\to F}(T) = -RT \ln K_{eq}^{U\to F}} \quad \text{(Eq. S3)}$$

where $p_{free}$ refers to the population of the unbound state and $p_B$ to the bound/'folded' state.

The linear dependence of the Gibbs free energy data obtained from Eq. S2 and S3 are explicitly described by Eq. S4.

$$\mathbf{\Delta G(T) = \Delta H^0 - T\Delta S^0} \quad \text{(Eq. S4)}$$

To described the non-linear dependence a further parameter needs to be introduced, namely a heat capacity ($C_P$) (Eq. S5)(7) describing the temperature dependence of enthalpy (Eq. S6).

$$\mathbf{\Delta C_P = \left(\frac{\delta H}{\delta T}\right)_P} \quad \text{(Eq. S5)}$$

$$\mathbf{\Delta G(T) = \Delta H_{T_m} - T\Delta S_{T_m} + \Delta C_P(T - T_m) - T\Delta C_P \ln\left(\frac{T}{T_m}\right)} \quad \text{(Eq. S6)}$$

ΔH(T) and ΔS(T) was determined according to Eq. S7 and S8 with a reference temperature of $T_m$.

$$\mathbf{\Delta H(T) = \Delta H(T_m) + \Delta C_P(T - T_m)} \quad \text{(Eq. S7)}$$

$$\mathbf{\Delta S(T) = \Delta S(T_m) + \Delta C_P \ln(T/T_m)} \quad \text{(Eq. S8)}$$

Here, $\mathbf{T_m = \Delta H^0/\Delta S^0}$ was determined from a linear fit of the three highest temperatures (281-287 K).

## FIBRILLIZATION KINETICS MONITORED BY pFTAA

We incubated 20 μM $A\beta_{40}$ and 5 μM $A\beta_{42}$ at +37 °C under quiescent conditions in the presence of different Ag(I) concentrations and samples supplemented with pFTAA and ThT were measured simultaneously for comparison (Fig. S1C-F). In the presence of pFTAA we observed the typically sigmoidal aggregation kinetic traces of $A\beta_{40}$ and $A\beta_{42}$(8, 9), while the aggregation behavior reported by ThT is less conclusive and exhibits a great increase in fluorescence signal at high Ag(I) concentrations for $A\beta_{40}$ of about two-fold (Fig. S1D,F). To investigate whether the increase in ThT fluorescence intensity may correspond to higher amounts of amyloid material, we performed CD spectra of the samples after the fibrillization experiments, supplemented with either pFTAA or ThT (Fig. S1G,H). All samples showed β-structures with similar intensities, regardless of the amyloid-detecting probe or Ag(I) ion concentration, indicating the same amount and structural state of the aggregated material in the presence of pFTAA and ThT. The increase in fluorescence intensity should thus originate from interaction of ThT and Ag(I) ions at high concentrations. Indeed, in literature silver ions have been reported to interact and affect the properties of ThT molecules, *e.g.* Ag(I) ions and silver nanoparticles have been shown to increase the ThT fluorescence yield(10, 11) and due to this property ThT can be used to detect Ag(I) ions in water(12). This process hence makes ThT unsuitable as a monitoring dye for protein fibrillization experiments in the presence of high Ag(I) concentrations. While the use of ThT is well established in protein kinetics assays(9, 13), we tested whether the presence of pFTAA interferes with the fibrillization process (Fig. S2). We recorded aggregation kinetic traces of 8 μM $A\beta_{42}$ in the presence of different concentrations of pFTAA and found that the final fluorescence intensity increases linearly with increasing pFTAA concentration (Fig. S2B). The aggregation kinetics are however only slightly affected and the effect of pFTAA is small compared to the variability of the replicates and particularly to the effect of Ag(I) (Fig. S2). We hence conclude that pFTAA is a suitable agent to monitor Aβ aggregation kinetics in the presence of Ag(I) and used it in all aggregation kinetics experiments described here.

## INTERACTION STUDIES USING CD

Interactions of $A\beta_{40}$ and Ag(I) were also investigated using circular dichroism (CD) where Ag(I) was titrated onto a monomeric 10 μM $A\beta_{40}$ sample (Fig. S11B). The CD spectrum without Ag(I) showed a predominantly unstructured state(14, 15) and upon addition of low to equimolar Ag(I) concentrations the spectra remained basically unchanged. These results indicate thus that the secondary structure content of $A\beta_{40}$ is not significantly altered upon binding of Ag(I), which is in line with the small chemical shift changes observed by NMR.

## THERMODYNAMICS OF THE Ag(I)-BINDING REACTION

The $p_B$ parameter is temperature dependent and related to the Gibbs free energy difference, $\Delta G_{U\to F}$ between the free monomer (unfolded state) and the bound/'folded' state via the equilibrium constant. Here, we apply a two-state model. The equilibrium constant between the 'folded'/bound and the free state, $K_{eq}^{U\to F}$ then determines the Gibbs free energy difference by $\Delta G_{U\to F}(T)$, which is displayed in Fig. 3H and listed in Table S4. Interestingly, when considering the full temperature range the values of $\Delta G_{U\to F}(T)$ revealed a non-linear behavior, which translates to non-negligible temperature dependences of the enthalpy, ΔH, and entropy, ΔS, differences. For higher temperatures (281-287 K), the values exhibit a good linear dependence, delivering the temperature-independent ΔH° and ΔS° (Table S6). Both the enthalpy and the entropy difference have negative

values, indicating a favorable Ag(I)-induced folding favorable in terms of enthalpy but disfavored in terms of entropy.

The non-linear temperature dependence of $\Delta G_{U \to F}(T)$ can generally be described introducing a heat capacity difference, $\Delta C_P$, between the folded and unfolded state. Applying this dependence, the change in heat capacity change $\Delta C_P$ was determined to -8.8 kJ mol$^{-1}$ K$^{-1}$, which is in the same range as previous results with A$\beta_{40}$ and Zn(II) ions(2) (Table S7). Loss of coordinated water molecules around the peptide can cause negative $\Delta C_P$ values and this may be the origin of the observed behavior. The observed temperature dependence can be described as cold denaturation of the protein folding reaction, which implies that the bound/'folded' state is never the major state and the 'folded' state thus is only marginally stable.

## TRANSIENT BINDING OF Ag(I) TO MONOMERIC Aβ STABILIZED BY SDS MICELLES

To test whether monomeric Aβ binds Ag(I), we performed experiments in the presence of SDS micelles. The amphiphilic Aβ peptide readily interacts with hydrophobic surfaces, surfactants and lipid membranes(16, 17). Aβ peptides adopt different secondary structures in the presence of different SDS concentrations(18, 19). While sub-micellar concentrations promote β-structure formation, SDS micelles bind and stabilize monomeric Aβ, where the N-terminal part of the peptide is located outside the micelle. In the two hydrophobic patches α-helical structure is induced, which are bound to the hydrophobic parts of the micelle(20). This system thus facilitates study of metal ion binding to the solvent-accessible N-terminus, while the SDS micelles constrain the peptide in a monomeric conformation. We followed the effect of 1:5 Ag(I):A$\beta_{40}$ on 170 μM A$\beta_{40}$ in SDS micelles with $^{1}$H-$^{15}$N-HSQC experiments and found that similar ligands in the SDS-bound Aβ peptide as in buffer solution are involved in coordinating the Ag(I) ion (Fig. S10A-C). Measurement of relaxation dispersion of this system revealed that three residues display strong relaxation dispersion profiles in the presence of SDS micelles at 298 K (Figs. S10D and S15). These three residues also show high relaxation dispersion amplitude without SDS micelles, yet the amount of data is not sufficient to perform a global fit analysis. These results hence support that indeed the N-terminus of monomeric A$\beta_{40}$ binds Ag(I).

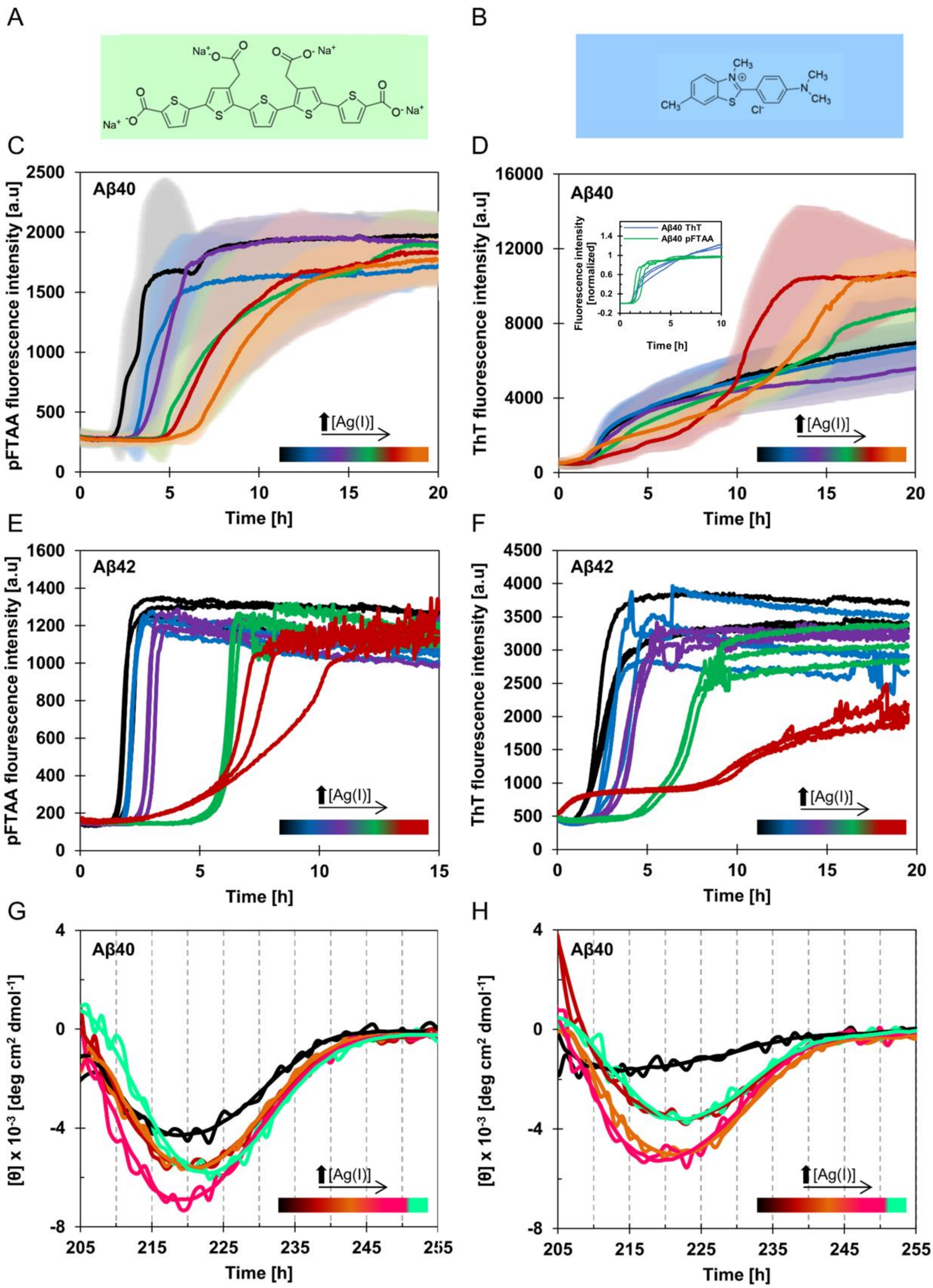


**Figure S1. Comparison pFTAA and ThT as amyloid-reporting dyes for Aβ$_{40}$ and Aβ$_{42}$ fibrillization kinetics in the presence of Ag(I) ions.** Molecular structures for pentameric formyl thiophene acetic acid (pFTAA) **(A)** and ThT **(B)**. **(C-F)** Kinetic traces following the fluorescence intensity of 20 µM Aβ$_{40}$ or 5 µM Aβ$_{42}$ incubated with Ag(I) ions (5, 10, 15, 20, 30 µM Ag(I) ions for Aβ$_{40}$ and 2.5, 5, 10, 50 µM Ag(I) ions for Aβ$_{42}$) in 10 mM MOPS buffer pH 7.2 at +37 °C under quiescent conditions in the presence of pFTAA **(C, E)** and ThT **(D, F)**. 0.3 µM pFTAA was used for both measurements with Aβ$_{40}$ and Aβ$_{42}$, while 40 µM ThT was

used for Aβ$_{40}$ and 10 μM ThT for Aβ$_{42}$. The kinetic data in **(C)** was used for further global fit analysis in the main manuscript in Fig. 1. Data presented for Aβ$_{40}$ or Aβ$_{42}$ were measured simultaneously on the same plate using peptides from the same stock solution. The inserted graph in **(D)** shows a comparison of pFTAA and ThT for Aβ$_{40}$ samples in the absence of Ag(I) ions from the kinetics curves in **(C)** and **(D)**. Samples from the end of the fibrillization kinetics experiments shown in **(C)** or **(D)** were taken for further measurements with circular dichroism (CD) spectroscopy. **(G, H)** CD spectra from samples incubated with Ag(I) ions (0, 20, 30, 50, 200 μM Ag(I) ions) supplemented with pFTAA **(G)** and ThT **(H)**, shown as smoothed data (10x smoothing).

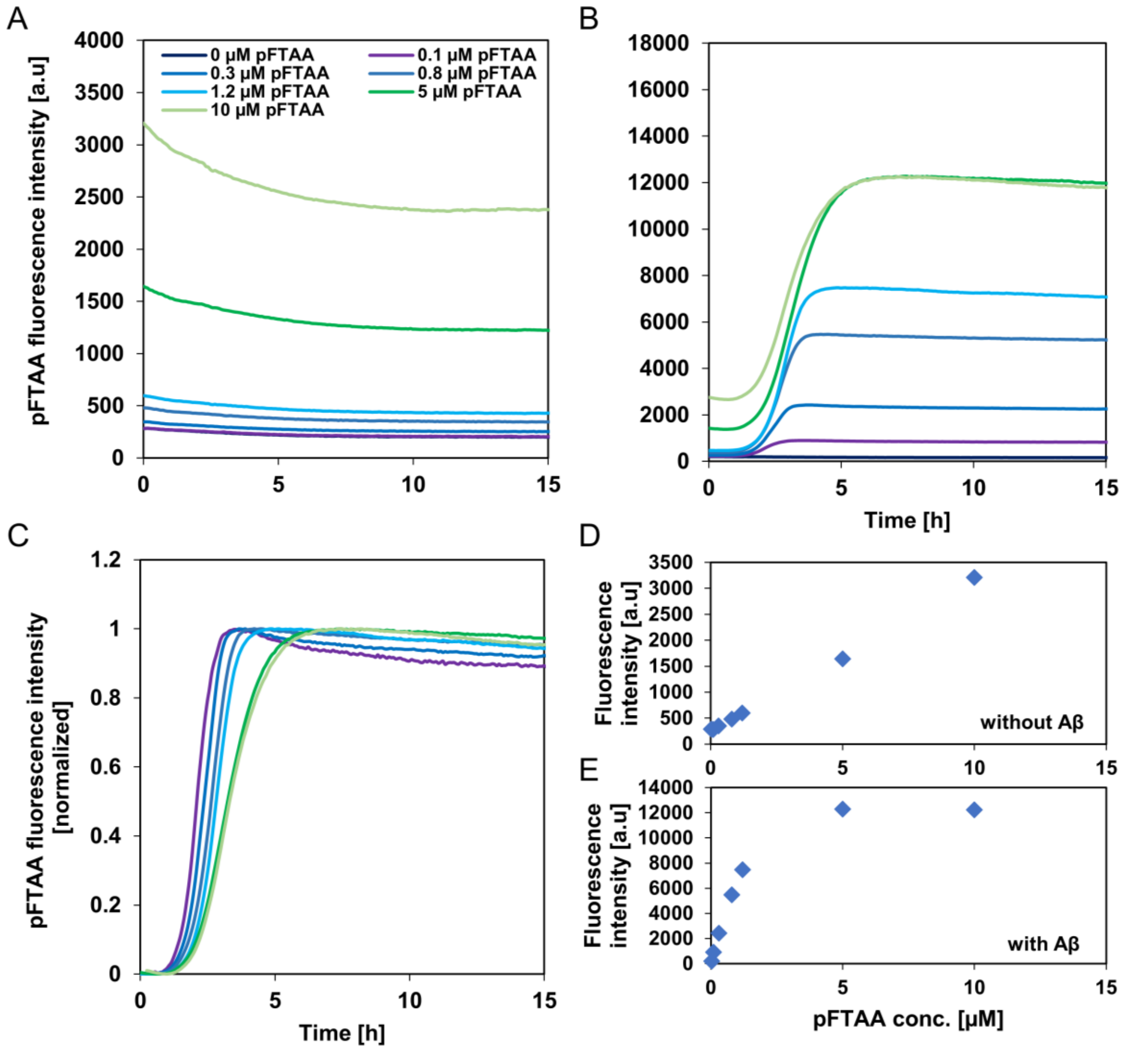


**Figure S2. Control experiments with different concentrations of pFTAA.**
Different concentrations of pFTAA (0-10 μM) were incubated in the absence **(A)** and presence of 8 μM Aβ$_{42}$ **(B, C)** in 10 mM MOPS buffer pH 7.8 at +37 °C under quiescent conditions. The data in **(C)** represents the normalized data presented in **(B)**. The end point fluorescence intensities versus pFTAA concentration in the absence of 8 μM Aβ$_{42}$ are presented in **(D)**, and in the presence of 8 μM Aβ$_{42}$ in **(E)**. Eight replicates were measured for each condition.

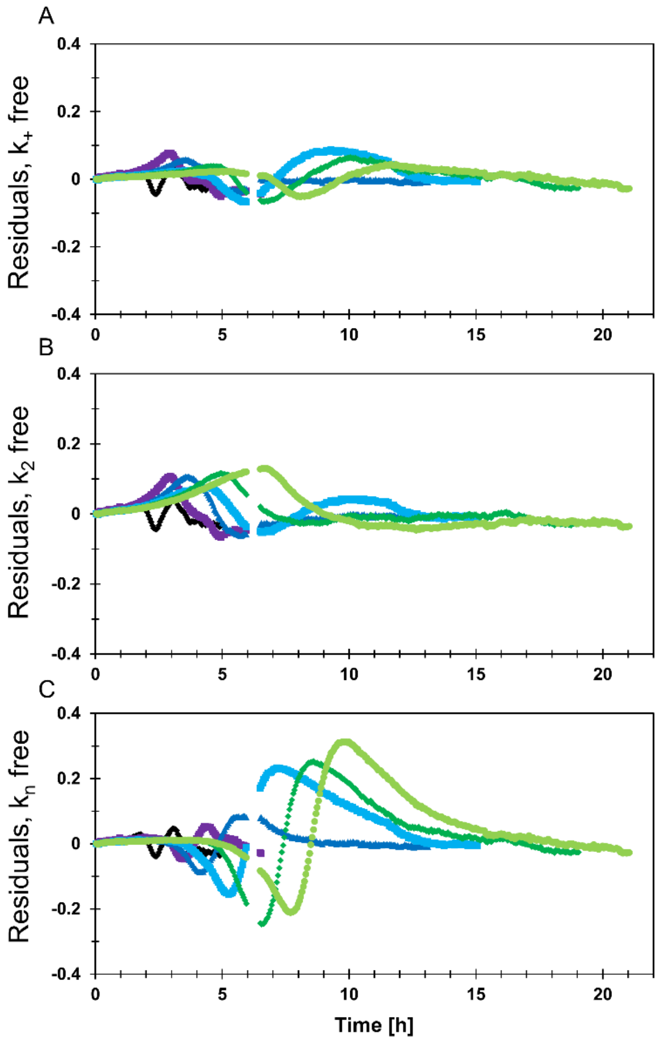


**Figure S3. Residuals from the global fit using 20 µM Aβ$_{40}$ peptides incubated with Ag(I) ions (0, 5, 10, 15, 20, 30 µM Ag(I) ions) presented in Fig. 1 in the main manuscript.** One rate constant, *i.e.* either $k_+$ in **(A)**, $k_2$ in **(B)** or $k_n$ in **(C)**, was allowed to vary while the other two rate constant parameters were held fixed across all Ag(I) concentrations.

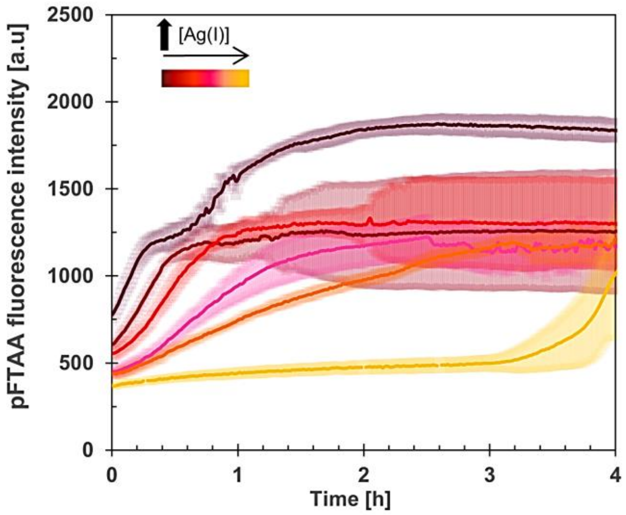


**Figure S4. Raw data from seeding experiments shown in Fig. 1 in the main manuscript.** 20 µM monomeric A$\beta_{40}$ peptides incubated with Ag(I) ions (0, 5, 10, 15, 20, 30 µM Ag(I) ions) in the presence of 1 µM pre-formed seeds in 10 mM MOPS buffer pH 7.2 and 0.3 µM pFTAA at +37 °C under quiescent conditions.

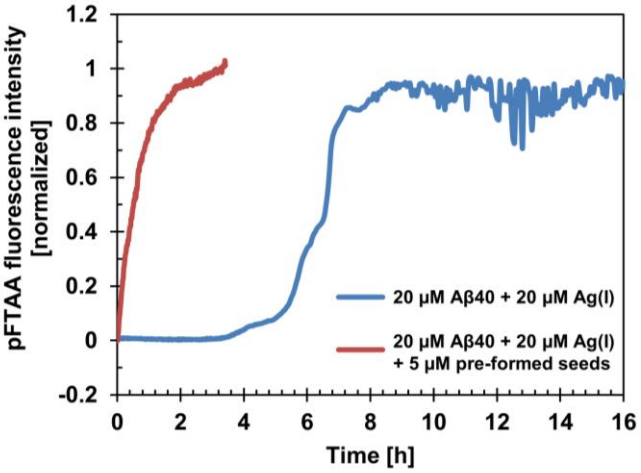


**Figure S5. Secondary nucleation is the dominant process for A$\beta_{40}$ peptide fibrillization in the presence of Ag(I) ions.**

20 µM monomeric A$\beta_{40}$ peptides were incubated in the presence of 20 µM Ag(I) ions in the absence and presence of 5 µM pre-formed seeds in 10 mM MOPS buffer pH 7.2 and 0.3 µM pFTAA at +37 °C under quiescent conditions. The seeded sample reached the plateau phase within the lag-time of the unseeded sample, indicating that secondary nucleation is the dominant mechanism also in the presence of Ag(I) ions.

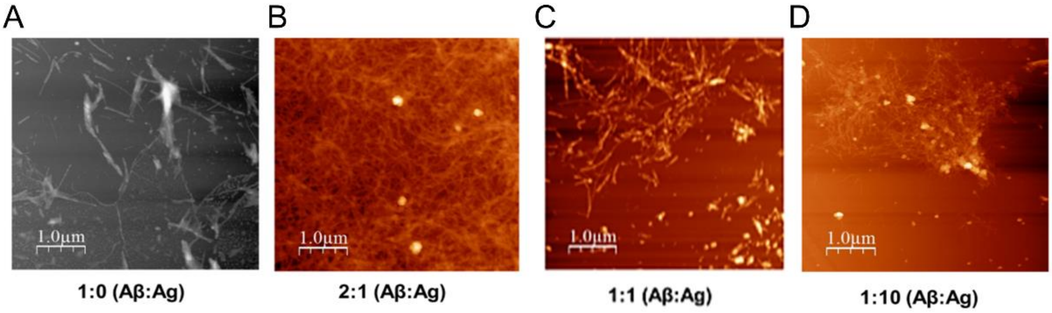


**Figure S6. Aβ fibrils formed in the absence and presence of Ag(I) ions indicate no obvious differences in fibril structure.**
Solid-state AFM images **(A-D)** from samples taken directly after fibrillization kinetic experiments with 15 μM fibrillated $A\beta_{40}$ peptides in 20 mM sodium phosphate buffer pH 7.4 and 40 μM ThT at +37 °C under quiescent conditions in the absence and presence of Ag(I) ions. Subfigures **(A)** and **(C)** are reprinted from Figure 1I and 1J in the main manuscript for direct comparison.

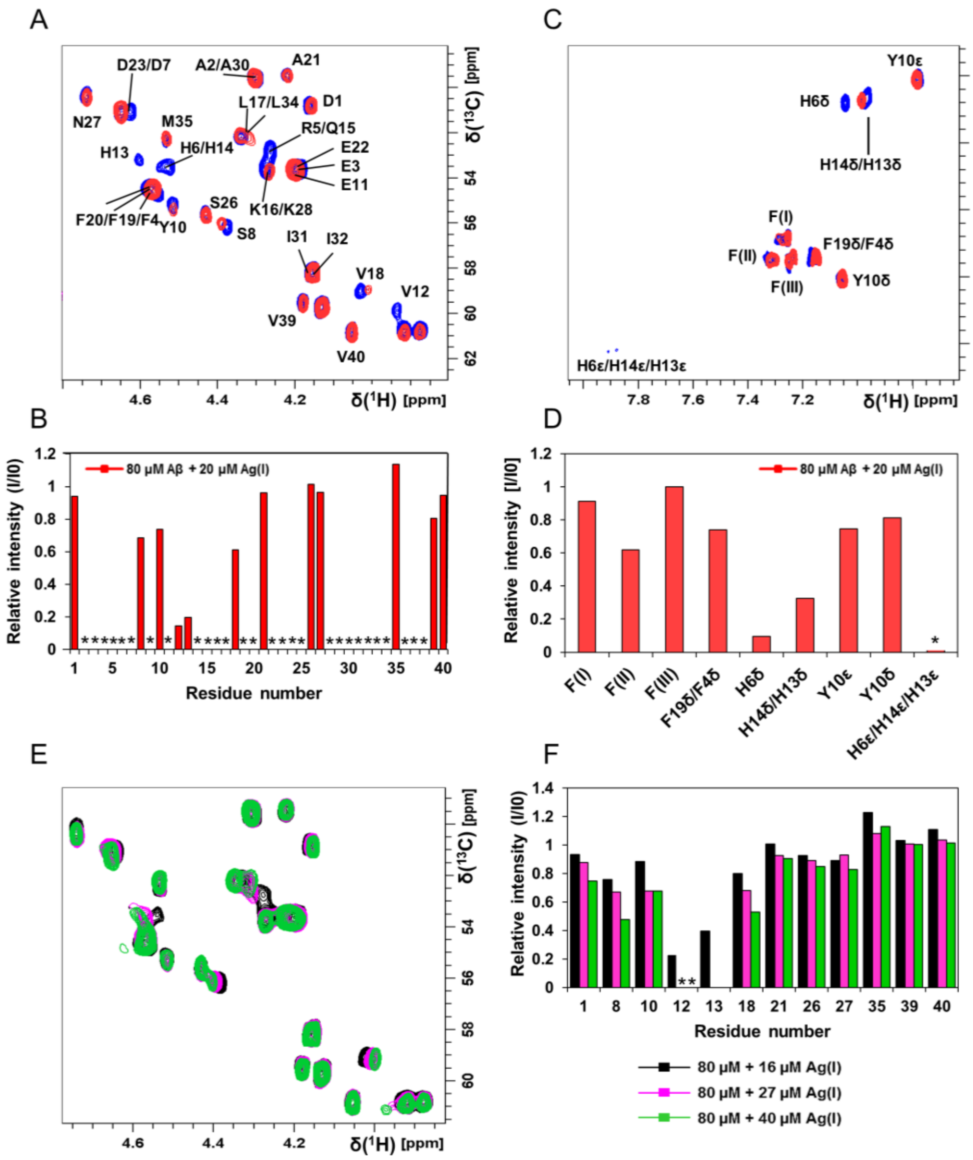


**Figure S7. Ag(I) ions bind specifically to the N-terminal part of the monomeric Aβ peptide inducing structural changes monitored by $^{1}$H-$^{13}$C-HSQC experiments.** Site-specific interaction information from 2D NMR $^{1}$H-$^{13}$C-HSQC experiments of 80 µM monomeric $^{13}$C-$^{15}$N-labeled Aβ$_{40}$ peptides in 20 mM sodium phosphate buffer pH 7.4 at 278 K recorded at a 700 MHz spectrometer equipped with a cryogenic probe **(A-F)**. **(A)** Spectra for the Cα-H region of Aβ$_{40}$ peptides alone (blue crosspeaks) and in the presence of 20 µM Ag(I) ions (red crosspeaks). The relative intensities from the spectra in **(A)** are shown in **(B)**, based on the amplitude of the crosspeaks with and without Ag(I) ions. **(C, D)** Spectra and relative intensities of the aromatic region in the presence of 20 µM Ag(I) ions (red crosspeaks). (**E, F**) Spectra and relative intensities for the same sample as in (**A-D**) are shown with further Ag(I) ion titration steps. **(E)** Spectra for the Cα-H region of Aβ$_{40}$ peptides in the presence of 16 µM Ag(I) ions (black crosspeaks), 27 µM Ag(I) ions (pink crosspeaks) and 40 µM Ag(I) ions (green crosspeaks). The relative intensities for the spectra in **(E)** are shown in **(F)**. Signal resonances assigned with an asterisk (*) are not shown due to low signal intensity and/or spectral overlap. The Aβ$_{40}$ crosspeak assignment in the HSQC spectra was performed by comparison with previously published work(21–23).

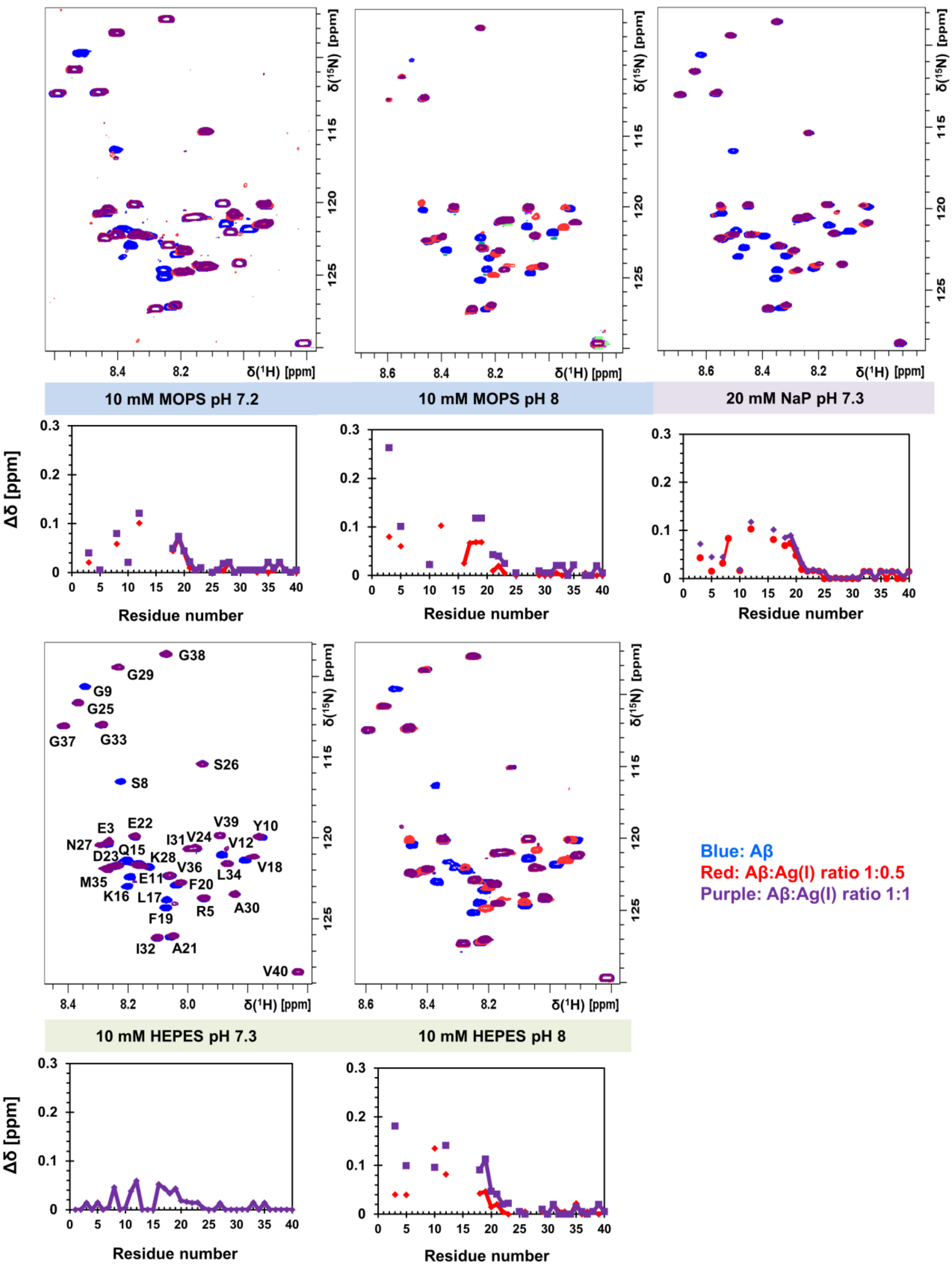


**Figure S8. Ag(I) ions bind specifically to the N-terminal part of the monomeric Aβ peptide in HEPES, MOPS and sodium phosphate buffer at physiological pH and at pH 8.**

Spectra and chemical shift differences from 2D NMR $^{1}H$-$^{15}N$-HSQC experiments of 80 μM monomeric $^{15}N$-labeled $A\beta_{40}$ peptides in 10 mM HEPES or MOPS buffer at 278 K recorded at a 500 MHz spectrometer with

a cryoprobe, or in 20 mM sodium phosphate buffer pH 7.3 at 278 K recorded at a 700 MHz spectrometer equipped with a cryogenic probe, are shown.

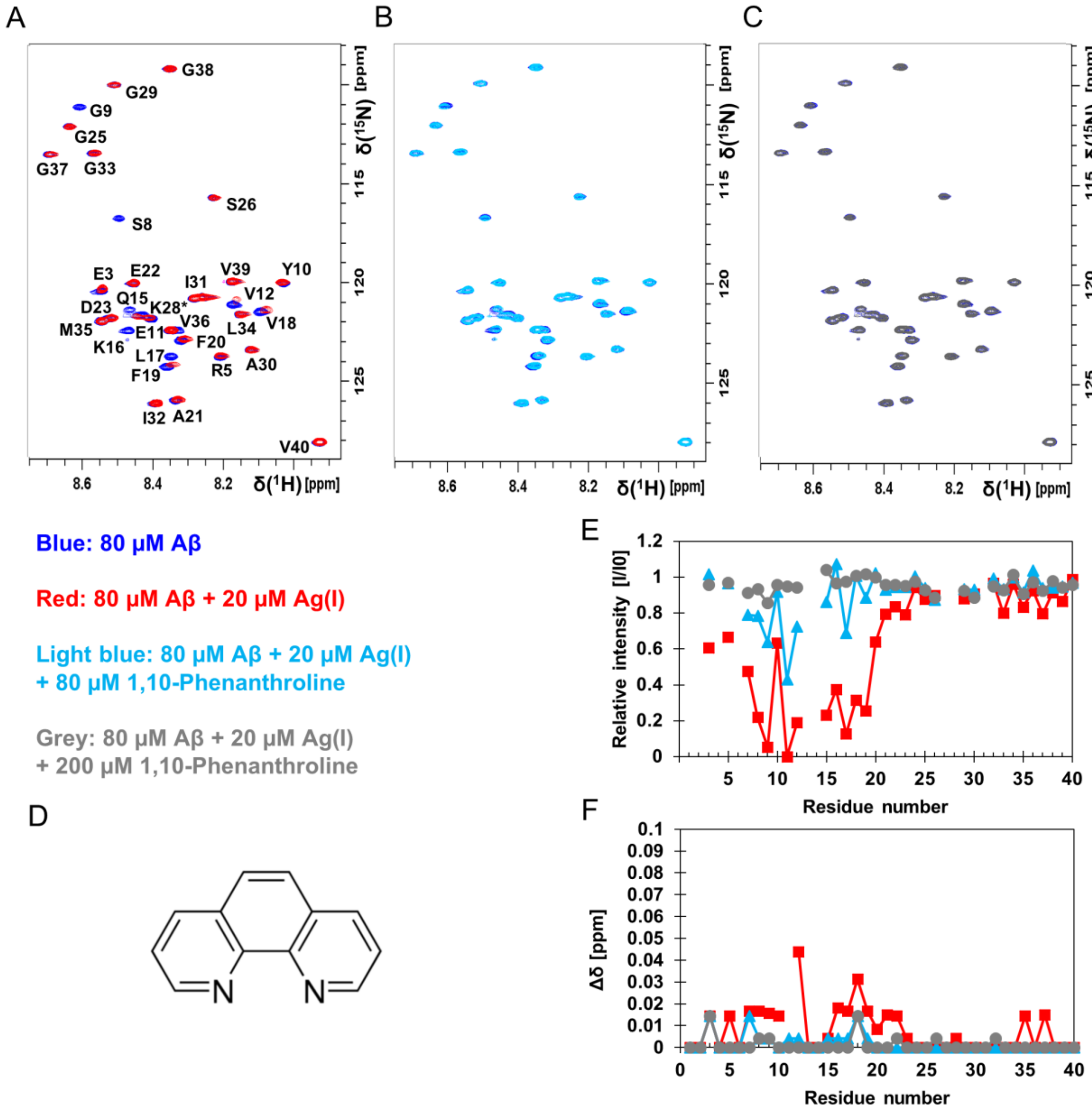


**Figure S9. Reversible binding of Ag(I) ions to the N-terminal part of the monomeric Aβ peptide probed with the 1,10-Phenanthroline chelator.**

Spectra and chemical shift differences from 2D NMR $^{1}H$-$^{15}N$-HSQC experiments of 80 µM monomeric $^{15}N$-labeled $A\beta_{40}$ peptides in 10 mM MOPS buffer pH 7.2 recorded at a 700 MHz spectrometer with a cryogenic probe at 278 K. **(A)** Spectra of 80 µM monomeric $^{15}N$-labeled $A\beta_{40}$ peptides alone (blue) and in the presence of 20 µM Ag(I) ions (red). Residues assigned with an asterisk (*) are not included in the analysis due to spectral overlap. **(B)** Spectra of 80 µM monomeric $^{15}N$-labeled $A\beta_{40}$ peptides alone (blue) and in the presence of 20 µM Ag(I) ions + **80 µM 1,10-Phenanthroline** (light blue). **(C)** Spectra of 80 µM monomeric $^{15}N$-labeled $A\beta_{40}$ peptides alone (blue) and in the presence of 20 µM Ag(I) ions + **200 µM 1,10-Phenanthroline** (grey). **(D)** The molecular structure of 1,10-Phenanthroline. The relative intensities and combined chemical shift changes from spectra in **(A-C)** are shown in **(E)** and **(F)**, respectively. The signal attenuation and chemical shift differences upon titration of Ag(I) ions are completely recovered in the presence of 200 µM 1,10-Phenanthroline.

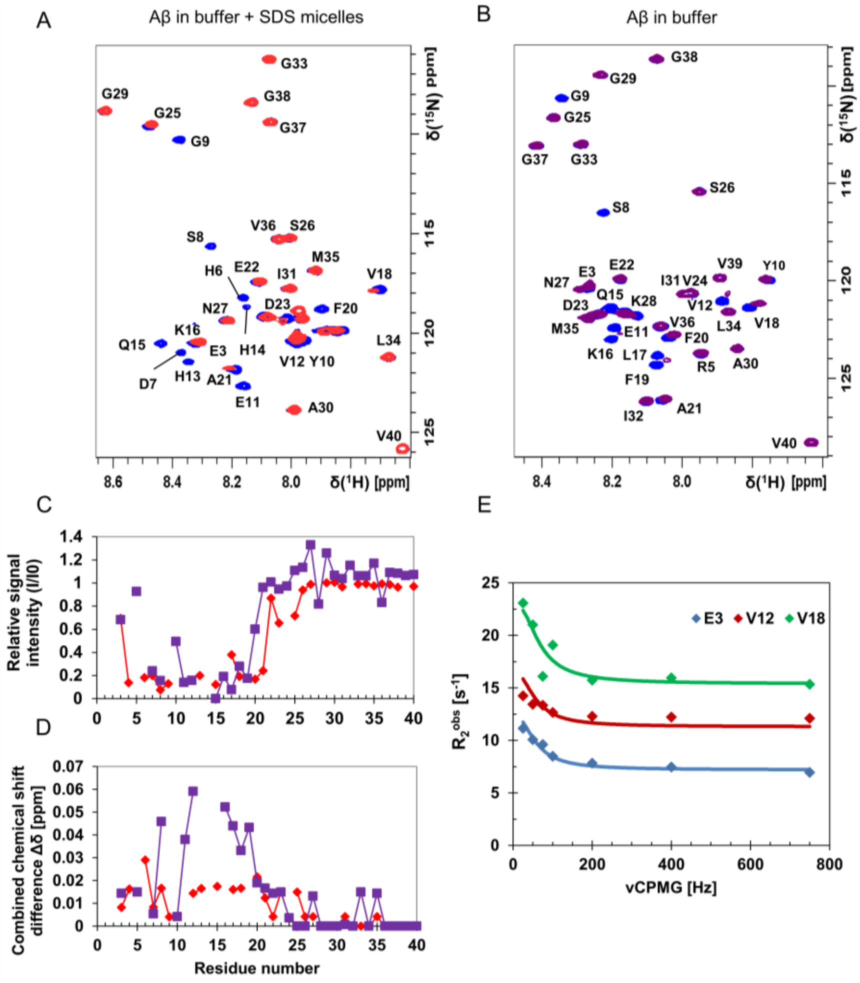


**Figure S10. 2D NMR HSQC and $^{15}$N-CPMG relaxation dispersion experiments, chemical exchange between free Aβ peptide and an Aβ-Ag(I) complex when the Aβ peptides are bound to SDS micelles.** **(A)** Spectra of 170 µM monomeric $^{15}$N-labeled $A\beta_{40}$ peptides, in 20 mM sodium phosphate buffer pH 7.4 and 50 mM d-SDS (>CMC), alone (blue) and in the presence of 34 µM Ag(I) ions (red) recorded at 700 MHz and 298 K. **(B)** Spectra of 80 µM monomeric $^{15}$N-labeled $A\beta_{40}$ peptides, in 10 mM HEPES buffer pH 7.2 **in the absence of d-SDS for comparison**, alone (blue) and in the presence of 80 µM Ag(I) ions (purple) recorded at 500 MHz and 278 K. **(C)** Relative intensities based on the amplitude of the amide crosspeaks with and without Ag(I) ions and **(D)** combined chemical shift changes. Red markers (♦) correspond to the spectra in **(A)** and purple markers (■) correspond to spectra shown in **(B)**. **(E)** Relaxation dispersion profiles from three N-terminal residues for the sample in **(A)**, showing significant chemical exchange.

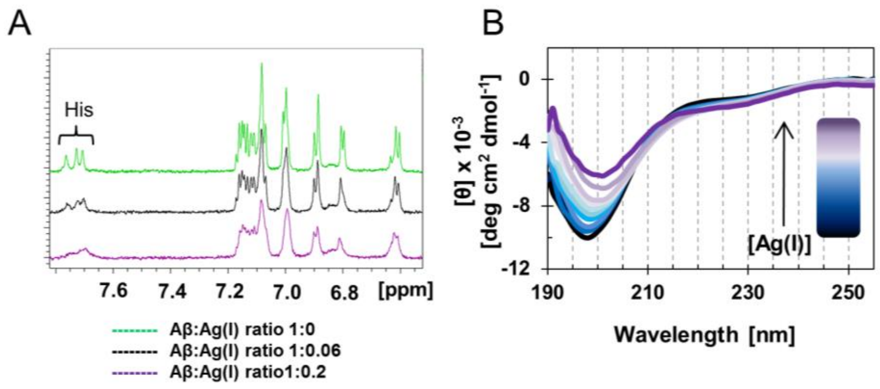


**Figure S11. Ag(I) ions bind specifically to histidine residues in the N-terminal part of monomeric Aβ peptide inducing local structural changes. (A)** 1D NMR experiments in $D_2O$ of $A\beta_{40}$ peptides in 10 mM

HEPES buffer pH 7.2 at 281 K confirm the observations of Ag(I) ion binding to A$\beta_{40}$ peptides as monitored by 2D NMR experiments. In the 1D spectra, the histidine signals are observed around 7.7 ppm, which are completely broadened upon addition of Ag(I) ions. **(B)** CD spectra were recorded to follow a titration of Ag(I) ions (1-1000 µM) onto 10 µM A$\beta_{40}$ in 20 mM sodium phosphate buffer pH 7.4 at 298 K. Intensity loss and small shifts towards longer wavelengths in the CD spectra were observed.

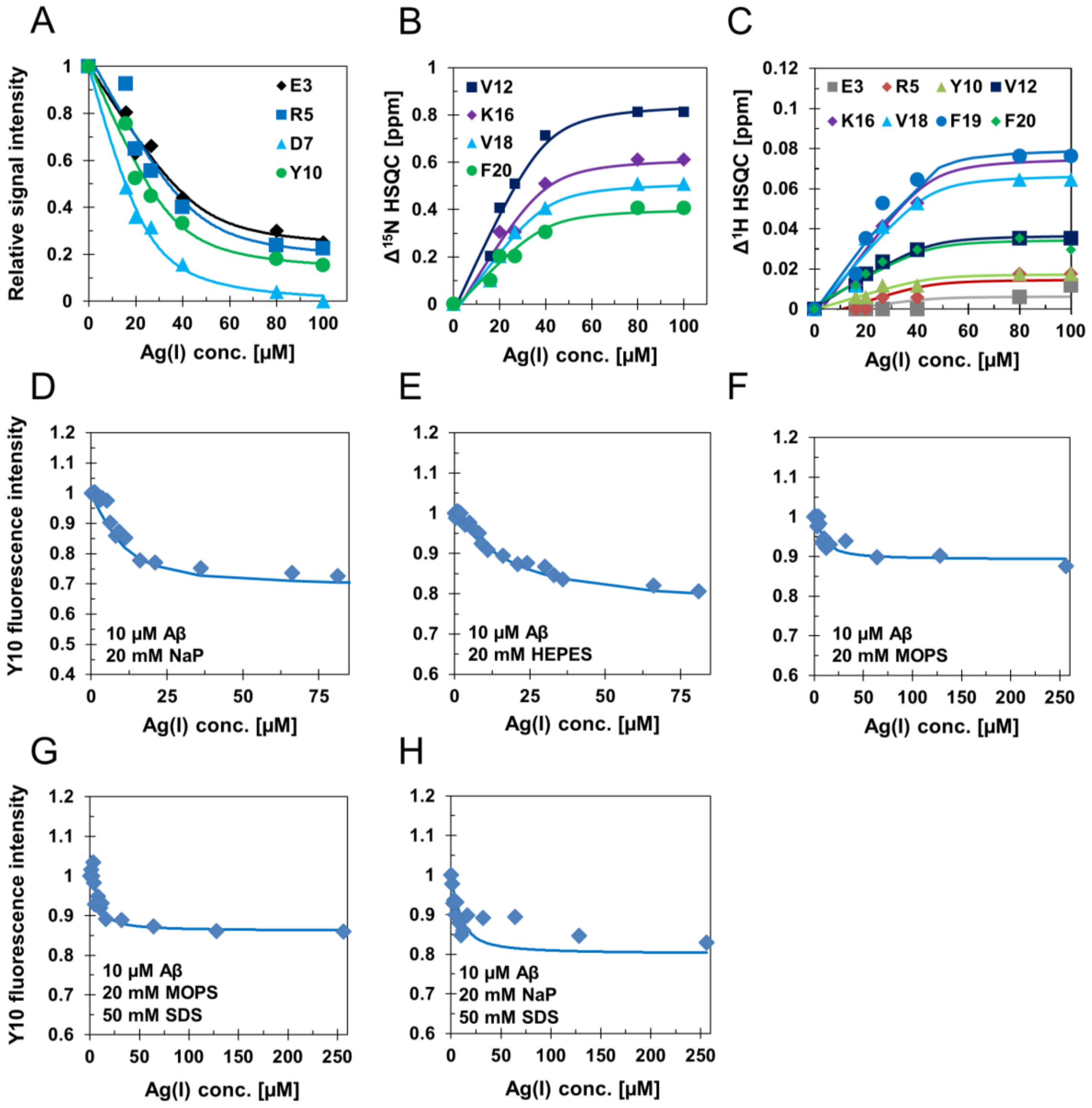


**Figure S12. Representative Ag(I) ion titration curves used for apparent dissociation constant ($K_D^{app}$) determination presented in Table S2**. **(A-C)** Titration curves obtained by 2D NMR $^1$H-$^{15}$N-HSQC experiments using a 80 µM $^{13}$C-$^{15}$N-labeled A$\beta_{40}$ sample in 20 mM sodium phosphate buffer pH 7.4 are shown. Residues selected for the global fitting in **(A)** were chosen based on gradual and clear signal attenuation upon Ag(I) ion titration. Titration curves using chemical shift changes ($\Delta^{15}$N and $\Delta^1$H) are presented in **(B)** and **(C)**, respectively. Selected residues in **(B)** and **(C)** were chosen based on clear chemical shift changes compared to the reference spectrum of Aβ in the absence of Ag(I) ions. Titration curves using Y10 intrinsic fluorescence data from 10 µM A$\beta_{40}$ in different buffers pH 7.4 and in the absence or presence of 50 mM SDS are shown in **(D-H)**. The titration experiments were performed in room temperature for Tyr10 fluorescence experiments and at 278 K for 2D NMR $^1$H-$^{15}$N-HSQC experiments. The determined dissociation constants are listed in Table S2.

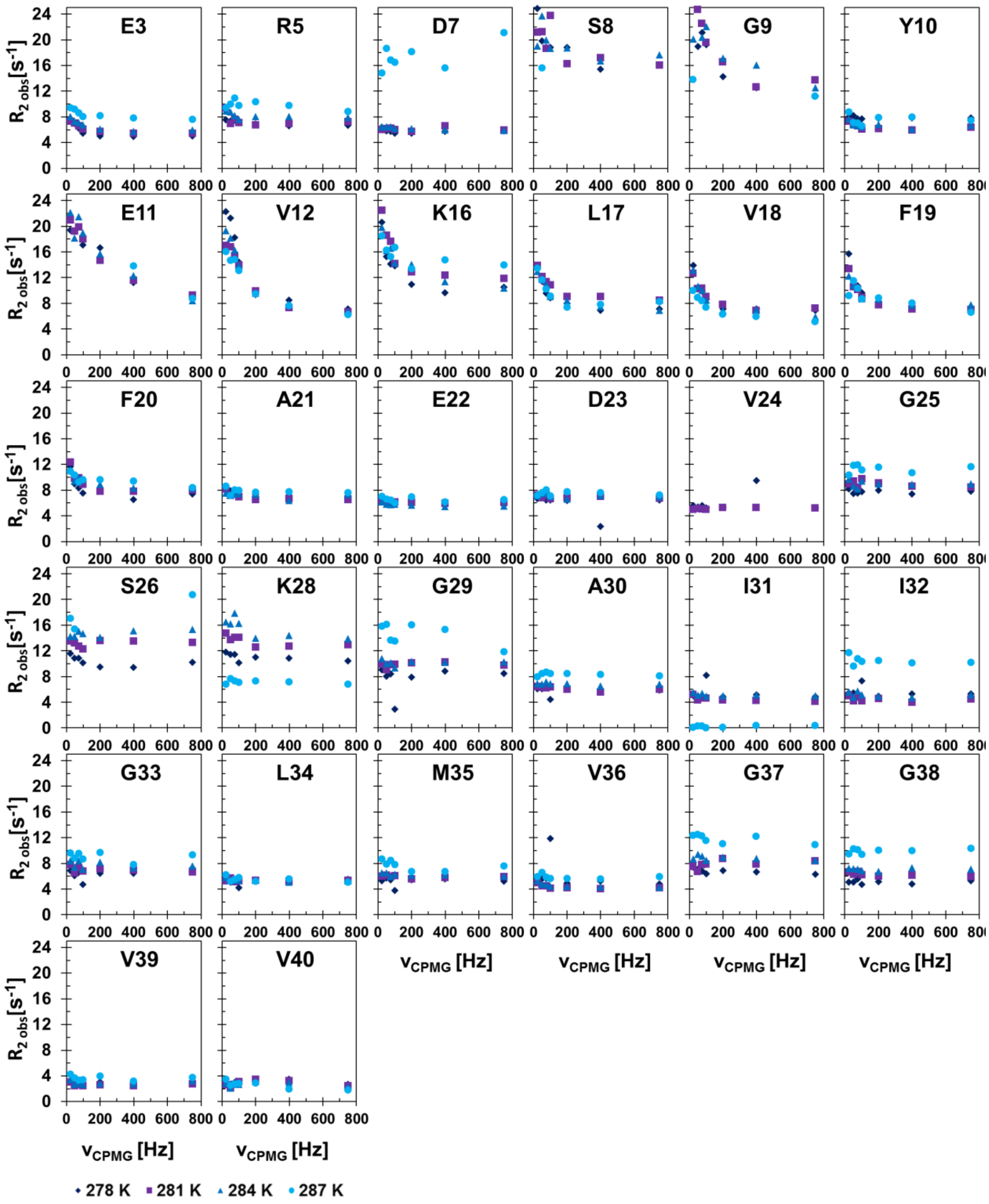


**Figure S13. $^{15}$N-CPMG relaxation dispersion profiles for all observable crosspeaks.** Data recorded with a 700 MHz spectrometer equipped with a cryoprobe and 80 µM monomeric $^{15}$N-labeled $A\beta_{40}$ peptides in 20 mM sodium phosphate buffer pH 7.4 and **4 µM Ag(I) ions** are shown.

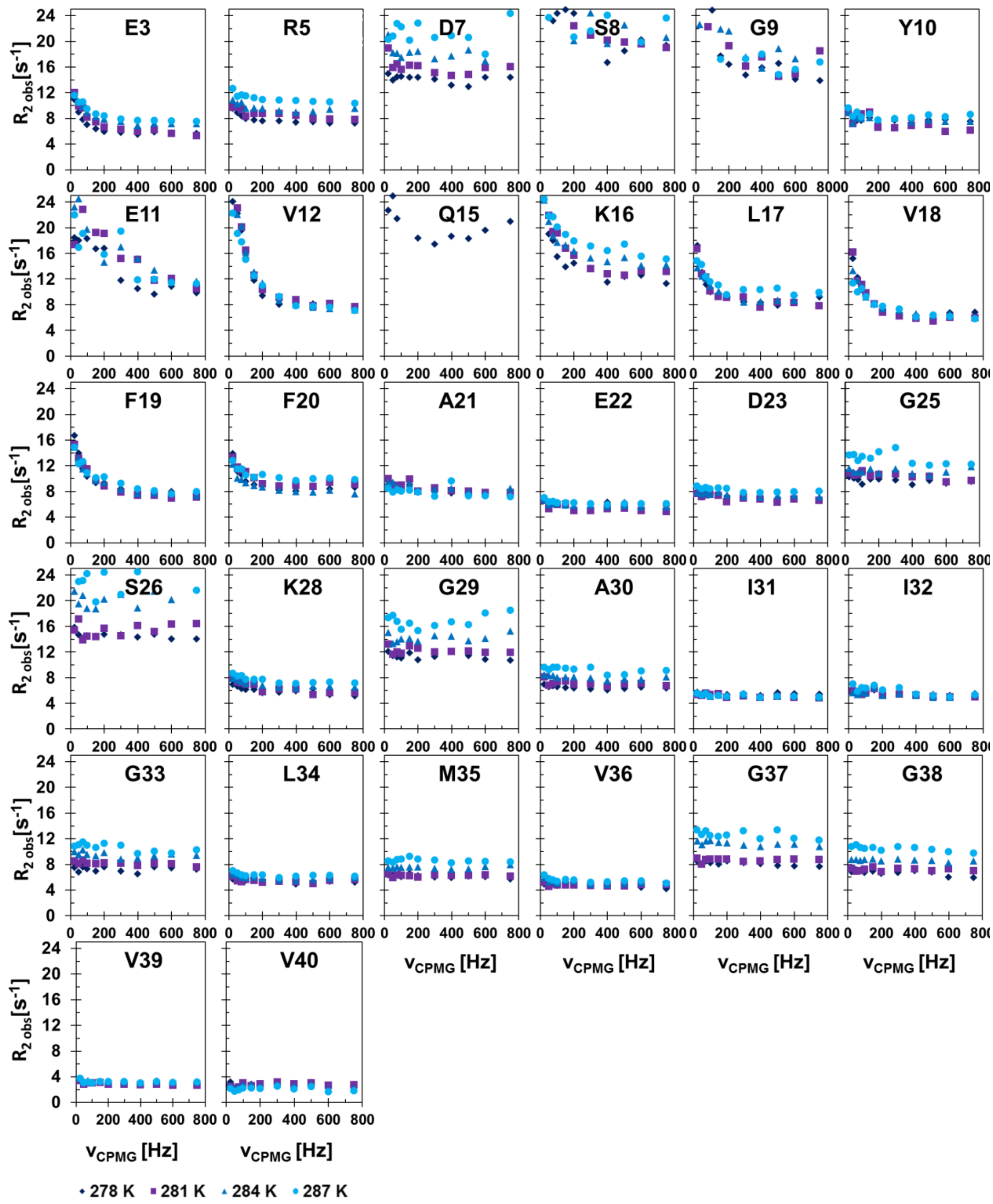


**Figure S14. $^{15}$N-CPMG relaxation dispersion profiles for all observable crosspeaks.** Data recorded with a 700 MHz spectrometer equipped with a cryoprobe and 80 µM monomeric $^{15}$N-labeled A$\beta_{40}$ peptides in 10 mM HEPES pH 7.4 and **6 µM Ag(I) ions** are shown.

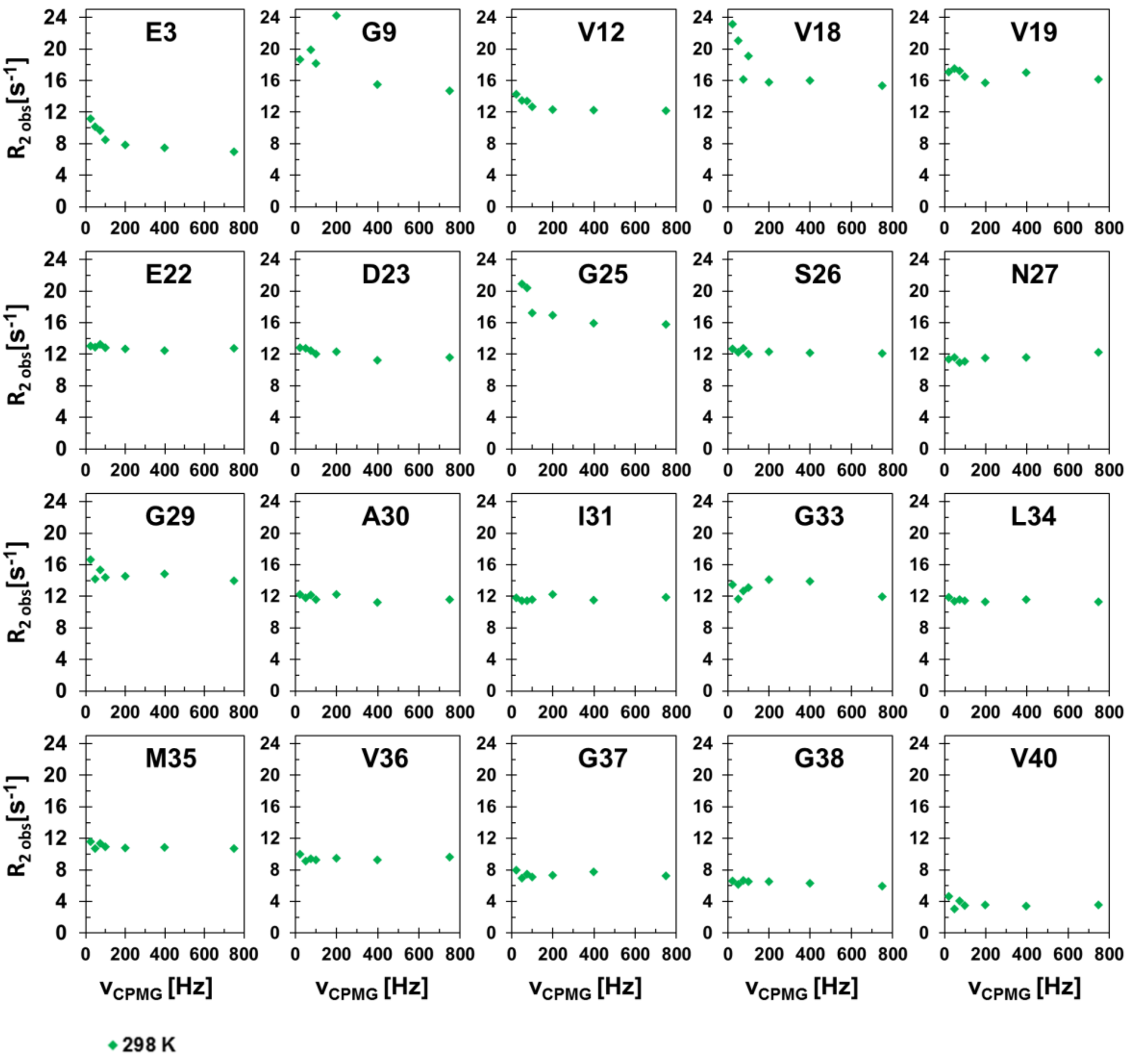


**Figure S15. $^{15}$N-CPMG relaxation dispersion profiles for all observable crosspeaks in SDS.** Data recorded with a 700 MHz spectrometer equipped with a cryoprobe and 170 µM monomeric $^{15}$N-labeled A$\beta_{40}$ peptides and **50 mM d-SDS** in 20 mM sodium phosphate buffer pH 7.4 and **34 µM Ag(I) ions** are shown.

**Table S1. Fundamental properties of silver-, zinc- and copper ions related to metal-protein coordination.**

| | **Ag(I)** | **Zn(II)** | **Cu(I)** | **Cu(II)** |
|---|---|---|---|---|
| | 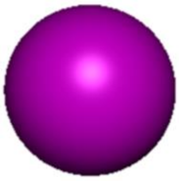 | 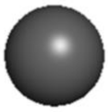 | 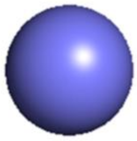 | 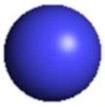 |
| **Pauling radius [pm]** | 126 (Ref. (24)) | 74 (Ref. (24)) | 96 (Ref. (24)) | 72 (Ref. (24)) |
| **Charge density [C mm$^{-3}$]** | 15 (Ref. (25)) | 112 (Ref. (25)) | 51 (Ref. (25)) | 116 (Ref. (25)) |
| **Lewis acid** | Soft (Ref. (26, 27)) | Intermediate (Ref. (27)) | Soft (Ref.(26, 27)) | Intermediate (Ref. (27)) |
| **Electron configuration** | [Kr]4d$^{10}$ | [Ar]3d$^{10}$ | [Ar]3d$^{10}$ | [Ar]3d$^{9}$ |
| **Coordination geometry** | linear, tetrahedral (Ref. (28)) | (not known) penta coordinated, tetra- or octahedral (Ref. (29, 30)) | Tetrahedral (Ref. (31)), diagonal (Ref. (32)), linear (Ref. (33, 34)) | distorted square planar (Ref. (34, 35)) |
| **Likely donor atoms** | SH<br>N<br>O (Ref. (36)) | 4N/O (Ref.(37),(38)) | SH (Ref. (27, 39))<br>2N (Ref. (38)) | 3N<br>>1 O (Ref. (38, 40)) |

**Table S2. Apparent dissociation constants ($K_D^{app}$) determined from Ag(I) ions titration experiments using Y10 intrinsic fluorescence effects or signal attenuation/chemical shift changes in 1D or 2D NMR $^1$H-$^{15}$N-HSQC experiments. Global fit analysis of kinetic curves where the apparent free Aβ monomer concentration is determined by the dissociation constant was also performed to obtain a $K_D^{app}$ value.** The errors reflect the errors from the fit. The titration curves are shown in Fig. S12 and the global fit of the kinetics curves is shown in Figure 1H in the main manuscript.

| | $K_D^{app}$ [μM] |
|---|---|
| 10 μM $Aβ_{40}$, 20 mM NaP pH 7.4 RT (Y10) | 8.8 ± 3.0 |
| 10 μM $Aβ_{40}$, 20 mM HEPES pH 7.2 RT (Y10) | 16.2 ± 2.7 |
| 10 μM $Aβ_{40}$, 20 mM MOPS pH 7.4 RT (Y10) | 4.3 ± 2.7 |
| 10 μM $Aβ_{40}$, 20 mM MOPS pH 7.4 RT, 50 mM SDS (Y10) | 4.8 ± 5.0 |
| 10 μM $Aβ_{40}$, 20 mM NaP pH 7.4 RT, 50 mM SDS (Y10) | 3.1 ± 2.0 |
| 80 μM $^{15}$N,$^{13}$C-$Aβ_{40}$, 20 mM NaP pH 7.4 278 K (NMR, HSQC signal attenuation) | 3.5 ± 3.6 |
| 80 μM $^{15}$N,$^{13}$C-$Aβ_{40}$, 20 mM NaP pH 7.4 278 K (NMR, HSQC $^{15}$N-chemical shift) | 2.0 ± 1.1 |
| 80 μM $^{15}$N,$^{13}$C-$Aβ_{40}$, 20 mM NaP pH 7.4 278 K (NMR, HSQC $^1$H-chemical shift) | 1.3 ± 1.6 |
| 80 μM $^{15}$N,$^{13}$C-$Aβ_{40}$, 20 mM NaP pH 7.4 278 K (NMR, 1D signal attenuation) | 2.5 ± 3.5 |
| 20 μM $Aβ_{40}$, 10 mM MOPS pH 7.2 +37 °C (Global fit analysis of kinetic curves) | 14.5 ± 0.2 |

**Table S3. Global fit parameters from the $^{15}$N-CPMG relaxation dispersion data.** The residues were divided into two groups, one group showing significant relaxation dispersion profiles (marked in green, analyzed with Model 1a) and one group with non-significant relaxation dispersion profiles (marked in red).

| 6 µM Ag(I) | $R_2^{obs}$ [$s^{-1}$] | | Δδ [ppm] | | p-value from F-test 6 µM Ag 278 K | 6 µM Ag 281 K | 6 µM Ag 284 K | 6 µM Ag 287 K |
|---|---|---|---|---|---|---|---|---|
| | | | | | | | P<0.01 | P>0.01 |
| **1** | | | | | | | | |
| **2** | | | | | | | | |
| **3** | 5.2 | ± 0.2 | 0.51 | ± 0.02 | 0.0002 | 0.001 | 6.58E-09 | 5.37E-07 |
| **4** | | | | | | | | |
| **5** | 7.3 | ± 0.32 | 0.34 | ± 0.14 | 0.035 | 0.237 | 8.20E-06 | 0.054 |
| **6** | | | | | | | | |
| **7** | 13.9 | ± 0.3 | 0.39 | ± 0.13 | 0.109 | 0.378 | 0.998 | 0.102 |
| **8** | 19.8 | ± 0.3 | 1.22 | ± 0.19 | 0.108 | 0.637 | 0.481 | 0.947 |
| **9** | 14.0 | ± 0.4 | 1.92 | ± 0.30 | 0.159 | 0.873 | 0.044 | 0.429 |
| **10** | 7.6 | ± 0.3 | 0.27 | ± 0.17 | 0.582 | 0.11 | 0.213 | 0.525 |
| **11** | 11.7 | ± 0.3 | 3.49 | ± 0.49 | 0.397 | 0.586 | 0.202 | 0.369 |
| **12** | 7.3 | ± 0.3 | 1.16 | ± 0.04 | 1.17E-05 | 6.62E-06 | 2.14E-08 | 2.46E-08 |
| **13** | | | | | | | | |
| **14** | | | | | | | | |
| **15** | | | | | | | | |
| **16** | 12.1 | ± 0.2 | 0.78 | ± 0.02 | 0.005 | 0.225 | 0.0005 | 0.0004 |
| **17** | 8.3 | ± 0.2 | 0.57 | ± 0.02 | 5.64E-05 | 0.006 | 2.14E-05 | 0.002 |
| **18** | 6.3 | ± 0.2 | 0.62 | ± 0.02 | 0.001 | 0.003 | 1.45E-06 | 9.02E-07 |
| **19** | 7.9 | ± 0.2 | 0.61 | ± 0.02 | 0.0099 | 0.013 | 4.11E-07 | 1.53E-05 |
| **20** | 8.4 | ± 0.2 | 0.42 | ± 0.02 | 0.007 | 0.277 | 3.77E-05 | 0.003 |
| **21** | 7.8 | ± 0.3 | 0.27 | ± 0.17 | 0.011 | 0.77 | 0.784 | 0.532 |
| **22** | 6.0 | ± 0.3 | 0.23 | ± 0.19 | 0.291 | 0.11 | 0.265 | 0.16 |
| **23** | 7.4 | ± 0.3 | 0.20 | ± 0.21 | 0.754 | 0.758 | 0.999 | 0.648 |
| **24** | | | | | | | | |
| **25** | 9.5 | ± 0.3 | 0.19 | ± 0.22 | 0.086 | 0.975 | 0.749 | 0.904 |
| **26** | 14.3 | ± 0.3 | 0.39 | ± 0.13 | 0.085 | 0.996 | 0.253 | 0.837 |
| **27** | | | | | | | | |
| **28** | 5.6 | ± 0.3 | 0.22 | ± 0.20 | 0.559 | 0.268 | 0.67 | 0.052 |
| **29** | 11.2 | ± 0.3 | 0.16 | ± 0.25 | 0.982 | 0.71 | 0.897 | 0.853 |
| **30** | 6.3 | ± 0.3 | 0.20 | ± 0.22 | 0.951 | 0.739 | 0.137 | 0.401 |
| **31** | 5.4 | ± 0.3 | 0.15 | ± 0.28 | 0.999 | 0.424 | 0.658 | 0.695 |
| **32** | 5.4 | ± 0.3 | 0.20 | ± 0.21 | 0.985 | 0.799 | 0.815 | 0.945 |
| **33** | 7.2 | ± 0.3 | 0.18 | ± 0.24 | 0.814 | 0.833 | 0.943 | 0.687 |
| **34** | 5.2 | ± 0.3 | 0.20 | ± 0.22 | 0.693 | 0.255 | 0.977 | 0.09 |
| **35** | 6.1 | ± 0.3 | 0.55 | ± 0.12 | 0.997 | 0.783 | 0.969 | 0.997 |
| **36** | 4.7 | ± 0.3 | 0.18 | ± 0.23 | 0.985 | 0.001 | 0.952 | 0.418 |
| **37** | 8.0 | ± 0.3 | 0.16 | ± 0.25 | 0.446 | 0.995 | 0.998 | 0.837 |
| **38** | 6.5 | ± 0.3 | 0.14 | ± 0.29 | 0.999 | 0.897 | 0.981 | 0.987 |
| **39** | 3.0 | ± 0.3 | 0.16 | ± 0.26 | 0.547 | 0.536 | 0.203 | 0.035 |
| **40** | 2.6 | ± 0.3 | 0.18 | ± 0.23 | 0.985 | 0.98 | 0.937 | 0.96 |

**Table S4. Global fit parameters from $^{15}$N-CPMG relaxation dispersion data shown in the main manuscript in Fig. 3, and calculated dissociation constants ($K_D^{app}$) using Eq. 1 in the main manuscript and Gibbs free energy values ($\Delta G_{U\text{-->}F}$).**

| Ag(I) conc. [µM] | Temperature [K] | $k_{ex}$ [$s^{-1}$] | $p_F$ [%] | $K_D^{app}$ [µM] | $\Delta G_{U\text{->}F}$ [kJ $mol^{-1}$] |
|---|---|---|---|---|---|
| 6 | 278 | 242 ± 20 | 8.9 ± 0.4 | ~1 | 5.4 |
| 6 | 281 | 278 ± 35 | 9.1 ± 0.6 | ~1 | 5.4 |
| 6 | 284 | 430 ± 33 | 7.2 ± 0.5 | ~3 | 6.0 |
| 6 | 287 | 481 ± 62 | 6.1 ± 0.8 | ~17 | 6.5 |
| 4 | 278 | 277 ± 35 | 7.0 ± 0.5 | ~1 | 6.0 |
| 4 | 281 | 264 ± 48 | 5.7 ± 0.6 | ~1 | 6.6 |
| 4 | 284 | 368 ± 62 | 5.5 ± 0.6 | ~1 | 6.7 |
| 4 | 287 | 478 ± 156 | 4.0 ± 1.3 | ~1 | 7.6 |
| **6** + 4 combined# | 278 | 239 ± 21 | 8.7 ± 0.5 | ~1 | 5.4 |
| 6 + **4** combined# | | 277 ± 32 | 7.0 ± 0.5 | ~1 | 6.0 |
| **6** + 4 combined# | 281 | 278 ± 33 | 9.1 ± 0.6 | ~1 | 5.4 |
| 6 + **4** combined# | | 264 ± 56 | 5.7 ± 0.7 | ~1 | 6.6 |
| **6** + 4 combined# | 284 | 430 ± 41 | 7.2 ± 0.6 | ~3 | 6.0 |
| 6 + **4** combined# | | 368 ± 49 | 5.5 ± 0.5 | ~1 | 6.7 |
| **6** + 4 combined# | 287 | 480 ± 73 | 6.0 ± 0.9 | ~19 | 6.5 |
| 6 + **4** combined# | | 478 ± 126 | 4.0 ± 1.0 | ~1 | 7.6 |

# The data sets for 6 and 4 µM Ag(I) were analysed together, where the chemical shift difference was constrained to the same value for both Ag(I) concentrations.

**Table S5. Global fit of [15]N-CPMG relaxation dispersion data shown in the main manuscript in Fig. 3 with different constraints.** Two different two-state exchange models were compared by using the F-test and the Akaike information criterion (AIC) method. The AIC values confirm the results of the F-test indicating Model 1 as the preferred model.

| **Model*** | **Constraints** | **$p_B$ [%] for 6 µM Ag(I)** | **$p_B$ [%] for 4 µM Ag(I)** | **$k_{ex}$ [$s^{-1}$] for 6 µM Ag(I)** | **$k_{ex}$ [$s^{-1}$] for 4 µM Ag(I)** | **$\chi^2$** | **# fitting para-meters** | **p-value of F-test Model 1 vs. Model X** | **AIC value Model 1 vs. Model X** |
|---|---|---|---|---|---|---|---|---|---|
| **1** | $\Delta\delta \geq \Delta\delta$(HSQC) | 6.1 to 8.9 | 4.0 to 7.0 | 200-500 | 200-500 | 196 | 79 | -- | -- |
| **1b** | $\Delta\delta \geq \Delta\delta$(HSQC) ($k_{ex}$ Ag(I) conc. independent) | 6.1 to 7.8 | 4.0 to 7.0 | 300-600 (4 and 6 µM Ag(I) combined) | 300-600 (4 and 6 µM Ag(I) combined) | 197 | 75 | 0.7 | $\gtrsim 0$ |
| **2** | $p_B$ temperature-independent $\Delta\delta \geq \Delta\delta$(HSQC) | 8.5 | 6.1 | 300-800 | 350-950 | 223 | 73 | << 0.01 | << 0 |

**General constraints:*

1. $p_B$ and $k_{ex}$ were constrained globally to the same value for all residues
2. $\Delta\delta$ was constrained to the same value for all temperatures

**Table S6. Thermodynamic parameters for Ag(I)-induced folding of the N-terminus of A$\beta_{40}$.**

| | $\Delta H_0$ [kJ $mol^{-1}$]* | $\Delta S_0$ [kJ $mol^{-1}$ $K^{-1}$]* | $\Delta H_{Tm}$ [kJ $mol^{-1}$]** | $\Delta S_{Tm}$ [kJ $mol^{-1}$ $K^{-1}$]** | $\Delta C_P$ [kJ $mol^{-1}$ $K^{-1}$]** |
|---|---|---|---|---|---|
| Sample | | | | | |
| 6 µM Ag(I) | -50 ± 5 | -0.20 ± 0.02 | 230 ± 6.0 *(± 180)* | 0.8 ± 0.02 *(± 0.7)* | -8.8 (± *6.0*) |

* from $\Delta G(T) = \Delta H - T\Delta S$

** from $f(T) = \Delta H - T*\Delta S + (\Delta C_P*(T-T_m)) - (T*\Delta C_P*\ln(T/T_m))$; Errors in brackets correspond to a fitting with three free parameters, while the given errors reflect the fitting errors when $\Delta C_P$ was fixed to a constant value.

**Table S7. Biophysical Ag(I)-Aβ and Zn(II)-Aβ data comparison.**

| | **Ag(I)** (results from this study) | **Zn(II)** (references see below) |
|---|---|---|
| **Metal Aβ binding region** | 6-20 | 1-18 (Ref. (2)) |
| **Likely Aβ ligands** | H6<br>H13<br>H14 | D1 ($NH_2$ and/or $COO^-$)<br>H6<br>H13<br>H14 (Ref. (21, 29)) |
| **Possible Aβ ligands** | D1<br>(E11)<br>(K16) | E3<br>E11<br>D7<br>$H_2O$ (Ref. (29)) |
| **2D NMR chemical shift changes** | Yes, N-terminal residues | Not observed (Ref. (2, 21)) |
| **$A\beta_{40}$ hydrodynamic radius in metal-bound state (peptide:metal ratio of ~2:1) [Å]** | 16.3 | 15.9 (Ref. (2)) |
| **Translational diffusion coefficient in metal-bound state (peptide:metal ratio of ~2:1) [$m^2/s$]** | $7.0 \times 10^{-11}$ | $7.2 \times 10^{-11}$ (Ref. (2)) |
| **Specific metal binding in SDS micelles** | Yes | Yes (Ref. (2, 6)) |
| **Instant aggregation upon additions of metal ions (sub-stoichiometric)** | No | No |
| **$A\beta_{40}^{noHis}$ metal binding** | No | No (Ref. (37)) |
| **Impact on Aβ fibrillation** | Retardation | Retardation (Ref. (2)) |
| **Dominating fibrillation process in the presence of sub-stoichiometric metal ion concentrations** | Secondary nucleation processes | Secondary nucleation processes (Ref. (2)) |
| **Main nucleation process affected** | Elongation rate constant | Elongation rate constant (Ref. (2)) |
| **Critical metal concentration for retardation [µM] (for 20 µM $A\beta_{40}$ aggregation kinetics)** | 5-30 | 0.25-2.5 (Ref. (2)) |
| **Metal ion impact on fibril structure of ThT/pFTAA end-point samples (at sub-stoichiometric metal ion concentrations)**<br>- **Similar final fluorescence signal**<br>- **Similar CD spectra** | <br>Yes<br>Yes | <br>Yes (Ref. (2))<br>Yes (Ref. (2)) |
| **$K_{D,app}$ to monomeric peptides in buffer [µM]** | 3-15 | 1-20 (Ref. (40))<br>1-6 (Ref. (21)) |
| **$\Delta C_P$ [$kJ \cdot mol^{-1} \cdot K^{-1}$]** | -8.8 (± *6.0*) | -8.1±1.0 (Ref. (2)) |

## References


1. Xue, C., Lin, T. Y., Chang, D., and Guo, Z. (2017) Thioflavin T as an amyloid dye: Fibril quantification, optimal concentration and effect on aggregation. *R. Soc. Open Sci.* **4**, 160696

2. Abelein, A., Gräslund, A., and Danielsson, J. (2015) Zinc as chaperone-mimicking agent for retardation of amyloid β peptide fibril formation. *Proc. Natl. Acad. Sci.* **112**, 5407–5412

3. Klingstedt, T., Åslund, A., Simon, R. A., Johansson, L. B. G., Mason, J. J., Nyström, S., Hammarström, P., and Nilsson, K. P. R. (2011) Synthesis of a library of oligothiophenes and their utilization as fluorescent ligands for spectral assignment of protein aggregates. *Org. Biomol. Chem.* **9**, 8356–8370

4. Civitelli, L., Sandin, L., Nelson, E., Khattak, S. I., Brorsson, A. C., and Kågedal, K. (2016) The luminescent oligothiophene p-FTAA converts toxic Aβ1-42 species into nontoxic amyloid fibers with altered properties. *J. Biol. Chem.* **291**, 9223–9243

5. Hellstrand, E., Boland, B., Walsh, D. M., and Linse, S. (2010) Amyloid β-protein aggregation produces highly reproducible kinetic data and occurs by a two-phase process. *ACS Chem. Neurosci.* **1**, 13–18

6. Tiiman, A., Luo, J., Wallin, C., Olsson, L., Lindgren, J., Jarvet, J., Per, R., Sholts, S. B., Rahimipour, S., Abrahams, J. P., Karlström, A. E., Gräslund, A., and Wärmländer, S. K. T. S. (2016) Specific Binding of Cu(II) Ions to Amyloid-Beta Peptides Bound to Aggregation-Inhibiting Molecules or SDS Micelles Creates Complexes that Generate Radical Oxygen Species. *J. Alzheimer's Dis.* **54**, 971–982

7. Oliveberg, M., Tan, Y. J., and Fersht, A. R. (1995) Negative activation enthalpies in the kinetics of protein folding. *Proc. Natl. Acad. Sci.* **92**, 8926–8929

8. Meisl, G., Yang, X., Hellstrand, E., Frohm, B., Kirkegaard, J. B., Cohen, S. I. A., Dobson, C. M., Linse, S., and Knowles, T. P. J. (2014) Differences in nucleation behavior underlie the contrasting aggregation kinetics of the Aβ40 and Aβ42 peptides. *Proc. Natl. Acad. Sci.* **111**, 9384–9389

9. Cohen, S. I. A., Linse, S., Luheshi, L. M., Hellstrand, E., White, D. A., Rajah, L., Otzen, D. E., Vendruscolo, M., Dobson, C. M., and Knowles, T. P. J. (2013) Proliferation of amyloid-beta 42 aggregates occurs through a secondary nucleation mechanism. *Proc. Natl. Acad. Sci.* **110**, 9758–9763

10. Wang, Y., Geng, F., Xu, H., Qu, P., Zhou, X., and Xu, M. (2012) A label-free oligonucleotide based thioflavin-t fluorescent switch for Ag+detection with low background emission. *J. Fluoresc.* **22**, 925–929

11. Makarava, N., Parfenov, A., and Baskakov, I. V. (2005) Water-soluble hybrid nanoclusters with extra bright and photostable emissions: A new tool for biological imaging. *Biophys. J.* **89**, 572–580

12. Li, Y. Y., Zhang, M., Lu, L. F., Zhu, A., Xia, F., Zhou, T., and Shi, G. (2015) Ratiometric fluorescence detection of silver ions using thioflavin T-based organic/inorganic hybrid supraparticles. *Analyst.* **140**, 6108–6113

13. Biancalana, M., and Koide, S. (2010) Molecular mechanism of Thioflavin-T binding to amyloid fibrils. *Biochim. Biophys. Acta - Proteins Proteomics*. **1804**, 1405–1412

14. Danielsson, J., Jarvet, J., Damberg, P., and Gräslund, A. (2005) The Alzheimer β-peptide shows temperature-dependent transitions between left-handed 31-helix, β-strand and random coil secondary structures. *FEBS J.* **272**, 3938–3949

15. Abelein, A., Jarvet, J., Barth, A., Gräslund, A., and Danielsson, J. (2016) Ionic Strength Modulation of the Free Energy Landscape of Aβ40Peptide Fibril Formation. *J. Am. Chem. Soc.* **138**, 6893–6902

16. Butterfield, S. M., and Lashuel, H. a. (2010) Amyloidogenic protein-membrane interactions: Mechanistic insight from model systems. *Angew. Chemie - Int. Ed.* **49**, 5628–5654

17. Österlund, N., Kulkarni, Y. S., Misiaszek, A. D., Wallin, C., Krüger, D. M., Liao, Q., Mashayekhy Rad, F., Jarvet, J., Strodel, B., Wärmländer, S. K. T. S., Ilag, L. L., Kamerlin, S. C. L., and Gräslund, A. (2018) Amyloid-β Peptide Interactions with Amphiphilic Surfactants: Electrostatic and Hydrophobic Effects. *ACS Chem. Neurosci.* **9**, 1680–1692

18. Abelein, A., Kaspersen, J. D., Nielsen, S. B., Jensen, G. V., Christiansen, G., Pedersen, J. S., Danielsson, J., Otzen, D. E., and Gräslund, A. (2013) Formation of dynamic soluble surfactant-induced amyloid β peptide aggregation intermediates. *J. Biol. Chem.* **288**, 23518–23528

19. Wahlström, A., Hugonin, L., Perálvarez-Marín, A., Jarvet, J., and Gräslund, A. (2008) Secondary structure conversions of Alzheimer's Aβ(1-40) peptide induced by membrane-mimicking detergents. *FEBS J.* **275**, 5117–5128

20. Jarvet, J., Danielsson, J., Damberg, P., Oleszczuk, M., and Gräslund, A. (2007) Positioning of the Alzheimer Aβ(1-40) peptide in SDS micelles using NMR and paramagnetic probes. *J. Biomol. NMR*. **39**, 63–72

21. Danielsson, J., Pierattelli, R., Banci, L., and Gräslund, A. (2007) High-resolution NMR studies of the zinc-binding site of the Alzheimer's amyloid beta-peptide. *FEBS J.* **274**, 46–59

22. Yamaguchi, T., Matsuzaki, K., and Hoshino, M. (2011) Transient formation of intermediate conformational states of amyloid-β peptide revealed by heteronuclear magnetic resonance spectroscopy. *FEBS Lett.* **585**, 1097–1102

23. Roche, J., Shen, Y., Lee, J. H., Ying, J., and Bax, A. (2016) Monomeric Aβ1-40 and Aβ1-42 Peptides in Solution Adopt Very Similar Ramachandran Map Distributions That Closely Resemble Random Coil. *Biochemistry*. **55**, 762–775

24. Ahrens, L. H. (1952) The use of ionization potentials Part 1. Ionic radii of the elements. *Geochim. Cosmochim. Acta*. **2**, 155–169

25. Rayner-Canham, G. (2014) *Descriptive Inorganic Chemistry 6e*

26. Veronesi, G., Gallon, T., Deniaud, A., Boff, B., Gateau, C., Lebrun, C., Vidaud, C., Rollin-Genetet, F., Carrière, M., Kieffer, I., Mintz, E., Delangle, P., and Michaud-Soret, I. (2015) XAS Investigation of Silver(I) Coordination in Copper(I) Biological Binding Sites. *Inorg. Chem.* **54**, 11688–11696

27. Lemire, J. A., Harrison, J. J., and Turner, R. J. (2013) Antimicrobial activity of metals: Mechanisms, molecular targets and applications. *Nat. Rev. Microbiol.* **11**, 371–384

28. Fox, B. S., Beyer, M. K., and Bondybey, V. E. (2002) Coordination chemistry of silver cations. *J. Am. Chem. Soc.* **124**, 13613–13623

29. Faller, P. (2009) Copper and zinc binding to amyloid-β: Coordination, dynamics, aggregation, reactivity and metal-ion transfer. *ChemBioChem*. **10**, 2837–2845

30. Kepp, K. P. (2012) Bioinorganic Chemistry of Alzheimer's Disease. *Chem. Rev.* **112**, 5193–5239

31. De Ricco, R., Potocki, S., Kozlowski, H., and Valensin, D. (2014) NMR investigations of metal interactions with unstructured soluble protein domains. *Coord. Chem. Rev.* **269**, 1–12

32. Faller, P., Hureau, C., and La Penna, G. (2014) Metal ions and intrinsically disordered proteins and peptides: From Cu/Zn amyloid-β to general principles. *Acc. Chem. Res.* **47**, 2252–2259

33. Hureau, C., and Faller, P. (2009) Aβ-mediated ROS production by Cu ions: Structural insights, mechanisms and relevance to Alzheimer's disease. *Biochimie*. **91**, 1212–1217

34. Atrián-Blasco, E., Gonzalez, P., Santoro, A., Alies, B., Faller, P., and Hureau, C. (2018) Cu and Zn coordination to amyloid peptides: From fascinating chemistry to debated pathological relevance. *Coord. Chem. Rev.* **371**, 38–55

35. Alies, B., Hureau, C., and Faller, P. (2013) The role of metal ions in amyloid formation: General principles from model peptides. *Metallomics*. **5**, 183–192

36. Li, H., Siu, K. W. M., Guevremont, R., and Le Blanc, J. C. Y. (1997) Complexes of silver(I) with peptides and proteins as produced in electrospray mass spectrometry. *J. Am. Soc. Mass Spectrom.* **8**, 781–792

37. Alies, B., Conte-Daban, A., Sayen, S., Collin, F., Kieffer, I., Guillon, E., Faller, P., and Hureau, C. (2016) Zinc(II) Binding Site to the Amyloid-β Peptide: Insights from Spectroscopic Studies with a Wide Series of Modified Peptides. *Inorg. Chem.* **55**, 10499–10509

38. Rana, M., and Sharma, A. K. (2018) Cu and Zn interactions with Aβ peptides: consequence of coordination on aggregation and formation of neurotoxic soluble Aβ oligomers. *Metallomics*. 10.1039/C8MT00203G

39. Pearson, R. G. (1963) Hard and Soft Acids and Bases. *J. Am. Chem. Soc.* **85**, 3533–3539

40. Faller, P., and Hureau, C. (2009) Bioinorganic chemistry of copper and zinc ions coordinated to amyloid-β peptide. *Dalt. Trans.* 10.1039/b813398k
